# Nylon-6/chitosan core/shell antimicrobial nanofibers for the prevention of mesh-associated surgical site infection

**DOI:** 10.1186/s12951-020-00602-9

**Published:** 2020-03-18

**Authors:** Antonios Keirouz, Norbert Radacsi, Qun Ren, Alex Dommann, Guido Beldi, Katharina Maniura-Weber, René M. Rossi, Giuseppino Fortunato

**Affiliations:** 1 Laboratory for Biomimetic Membranes and Textiles, Empa, Swiss Federal Laboratories for Materials Science and Technology, Lerchenfeldstrasse 5, CH-9014 St. Gallen, Switzerland; 2 School of Engineering, Institute for Materials and Processes, The University of Edinburgh, Robert Stevenson Road, Edinburgh, EH9 3FB UK; 3 Laboratory for Biointerfaces, Empa, Swiss Federal Laboratories for Materials Science and Technology, Lerchenfeldstrasse 5, CH-9014 St. Gallen, Switzerland; 4 Center for X-Ray Analytics, Empa, Swiss Federal Laboratories for Materials Science and Technology, Überlandstrasse 129, CH-8600 Dübendorf, Switzerland; 5 Department of Visceral Surgery and Medicine, Visceral Surgery, Inselspital University Hospital Bern and University Bern, Freiburgstrasse 18, CH-3010 Bern, Switzerland

**Keywords:** Chitosan, Nylon-6, Co-axial electrospinning, Hernia meshes, Antimicrobial fibers, Drug release

## Abstract

The state-of-the-art hernia meshes, used in hospitals for hernia repair, are predominantly polymeric textile-based constructs that present high mechanical strength, but lack antimicrobial properties. Consequently, preventing bacterial colonization of implanted prosthetic meshes is of major clinical relevance for patients undergoing hernia repair. In this study, the co-axial electrospinning technique was investigated for the development of a novel mechanically stable structure incorporating dual drug release antimicrobial action. Core/shell structured nanofibers were developed, consisting of Nylon-6 in the core, to provide the appropriate mechanical stability, and Chitosan/Polyethylene oxide in the shell to provide bacteriostatic action. The core/shell structure consisted of a binary antimicrobial system incorporating 5-chloro-8-quinolinol in the chitosan shell, with the sustained release of Poly(hexanide) from the Nylon-6 core of the fibers. Homogeneous nanofibers with a "beads-in-fiber" architecture were observed by TEM, and validated by FTIR and XPS. The composite nanofibrous meshes significantly advance the stress–strain responses in comparison to the counterpart single-polymer electrospun meshes. The antimicrobial effectiveness was evaluated in vitro against two of the most commonly occurring pathogenic bacteria; *S. aureus* and *P. aeruginosa*, in surgical site infections. This study illustrates how the tailoring of core/shell nanofibers can be of interest for the development of active antimicrobial surfaces.

## Introduction

Hernia repair is one of the most commonly performed elective operations with approximately 100,000 hernia repair surgeries being carried out in the UK, over 700,000 in the US, and 1,100,000 inguinal and abdominal wall hernia surgeries in China every year [1, 2]. Inguinal hernia surgery is the most frequent accounting for over 75%, followed by epigastric and incisional at 15% and other forms 10% [3]. The majority of inguinal hernias occur in men (98%), with 30% of patients developing a second hernia on the opposite side of the groin [4]. Suture closures are recognized for having high recurrence rates, while synthetic and bioprosthetic meshes carry their own downsides, such as being heavyweight, which induces foreign body sensation, leading to fibrosis and tissue adhesion, post-surgical infection, etc. [5]. The use of hernia meshes reduces recurrence by 30–50% compared to suture repair [6, 7]. In general, intravenous and oral administration of prophylactic antibiotics does not ensure sufficient protection against surgical site infection [8].

Nanofibrous scaffolds, developed via the electrospinning process can be valuable towards the development of a biodegradable antimicrobial layer as the fibers formed are lightweight, can attain a large surface area per unit mass [9], present different morphologies [10], and via the co-axial electrospinning technique (Additional file 1: Figure S1), it is feasible to incorporate antimicrobial agents in a spatially-controlled bilayer format. The utilization of the co-axial electrospinning technique provides further advantages over single-needle electrospinning as selected properties can be combined in one fiber, e.g. (1) fabricate electrospun membranes that incorporate a hydrophilic surface within a hydrophobic core into a single core-sheath fiber, (2) encapsulate and protect sensitive substances from the outer environment by placing the drug within the core, (3) provide programmable release kinetics from the core of the fibers; e.g. to tailor for sustained release kinetics, while allowing (4) the controlled release of defined concentrations of pharmaceutical ingredients [11–13]. Further, by integrating a mechanically adapted polymer into the core of the fibers, it is also feasible to improve the mechanical behavior of the final construct.

Chitosan (CS, a partially deacetylated chitin) is an abundant in nature, polycationic polymer, composed through an extended number of β(1–4) linked glucosamine and *N*-acetyl glucosamine units, and it is considered a valued biocompatible material with bactericidal properties (Additional file 1: Figure S2a) [14, 15]. It carries three reactive functional sites, an amine and a secondary hydroxyl group at C-6, and a primary hydroxyl group at C-3 [16]. Solid chitosan fibers can act through a wide range of mechanisms against both Gram ( +) and (–) bacteria [17] and chitosan is considered as both a bactericidal and a bacteriostatic agent. Due to its polycationic structure, CS can permeabilize the cell wall of prokaryotes by forming ionic complexes with the negative charges found: (1) on the phospholipids and lipopolysaccharides present in the outer and inner membrane on Gram (–) bacteria, and (2) the teichoic acids linked to the peptidoglycan present in the cell wall of Gram ( +) bacteria [18]. Such interactions provoke internal osmotic imbalances, leakage of intracellular electrolytes, and other low molecular weight proteinaceous constituents, consequently inhibiting growth. Hydrolyzed products of microbial DNA and RNA, also negatively charged, can ultimately inhibit downstream transcription and translation [19].

Nylon-6 (Polyamide-6, PA6) is synthesized by the formation of free-radicals via thermal decomposition of the ϵ-caprolactam ring, followed by chain growth through ring-opening polymerization (Additional file 1: Figure S2b) [20]. PA6 has been widely used as surgical material for non-absorbable synthetic sutures, while PA6 and polyurethane are conjointly utilized as balloon material in angioplasty, due to the superior tensile strength of PA6 [21, 22]. PA6 has several advantageous characteristics, such as flexible functionalization possibilities and superior mechanical performance compared to many other polymer materials [23]. PA6 has been shown to carry good responses and increased stability in bodily fluids [24].

Poly (hexamethylene biguanide) (Polyhexanide, PHMB) is a well-known disinfectant of mucous membranes and wounds, increasingly appearing in a variety of products, such as an antiseptic in wound dressings (Additional file 1: Figure S2c) [25]. PHMB is a small molecule, with a characteristic carbon tail adjacent to a biguanide complex. PHMB's chemical structure closely resembles that of chlorhexidine, the most commonly used disinfectant and antiseptic of the skin prior to surgical operations [26]. At physiological pH, PHMB is polycationic due to the monoprotonation of each biguanide residue [27]. Similar to chitosan, PHMB can widely permeabilize bacterial membranes. PHMB can interact with acidic-phospholipids present in bacteria membranes, subsequently causing their disruption, while the tissue’s neutral phospholipids are only affected in a limited extent [28]. However, contrary to solid forms of chitosan, due to its small size (180–500 Da), PHMB is able to infiltrate the bacterial wall and intercept cell division by condensing the negatively charged chromosomes—a property that previously has not been considered and has been shown to be selective to prokaryotes with no adverse responses to mammalian cells [29]. PHMB is an effective antimicrobial agent against Gram ( +) and Gram (–) bacterial species with bactericidal activity near 100%, even at low concentrations (4 mg L^−1^) [30]. Recently, PHMB 0.3% w/v was set to be the maximum concentration allowed by the *Scientific Committee on Consumer Safety* (SCCS) of the European Union [31] and has been chosen for many wound dressing products available on the market, such as Suprasorb® [32].

5-Chloro-8-hydroxyquinoline (Cloxyquin, 5CLO8Q) is a derivative of quinoline and belongs to the bihalogenated 8-hydroxyquinolines family. 5CLO8Q is slightly soluble in water and has previously shown to be active against various bacteria, as well as fungal and amoebic organisms [33]. Quinolines are aromatic nitrogen compounds that present a bicyclic structure consisting of a saturated benzene ring being joint at two carbons with a pyridine ring (Additional file 1: Figure S2d). Hongmanee et al. tested the activity of 5CLO8Q against 150 strains of *Mycobacterium tuberculosis*, a species of pathogenic bacteria whose cell wall has features of both Gram ( +) and Gram (−) bacteria, which demonstrated good bactericidal responses [34]. Darby and Nathan found that 5CLO8Q had bactericidal activity against non-replicating and replicating *M. tuberculosis*, a feature that is lacking in the currently approved drugs on the market [35]. The mechanisms of action of 5CLO8Q in bacteria are poorly understood, but they are thought to relate to its chelating activities. It has been suggested that iron chelation deprives microorganisms of essential nutrients.^31^ Further, it has been proposed that it can inhibit the RNA-dependent DNA polymerase of respiratory syncytial viruses by chelation of copper and inhibit the synthesis of RNA by chelation of Mg^2+^, Zn^2+^, and Mn^2+^, a similar mechanism could apply for a bacterial species [36].

**Fig. 1:**
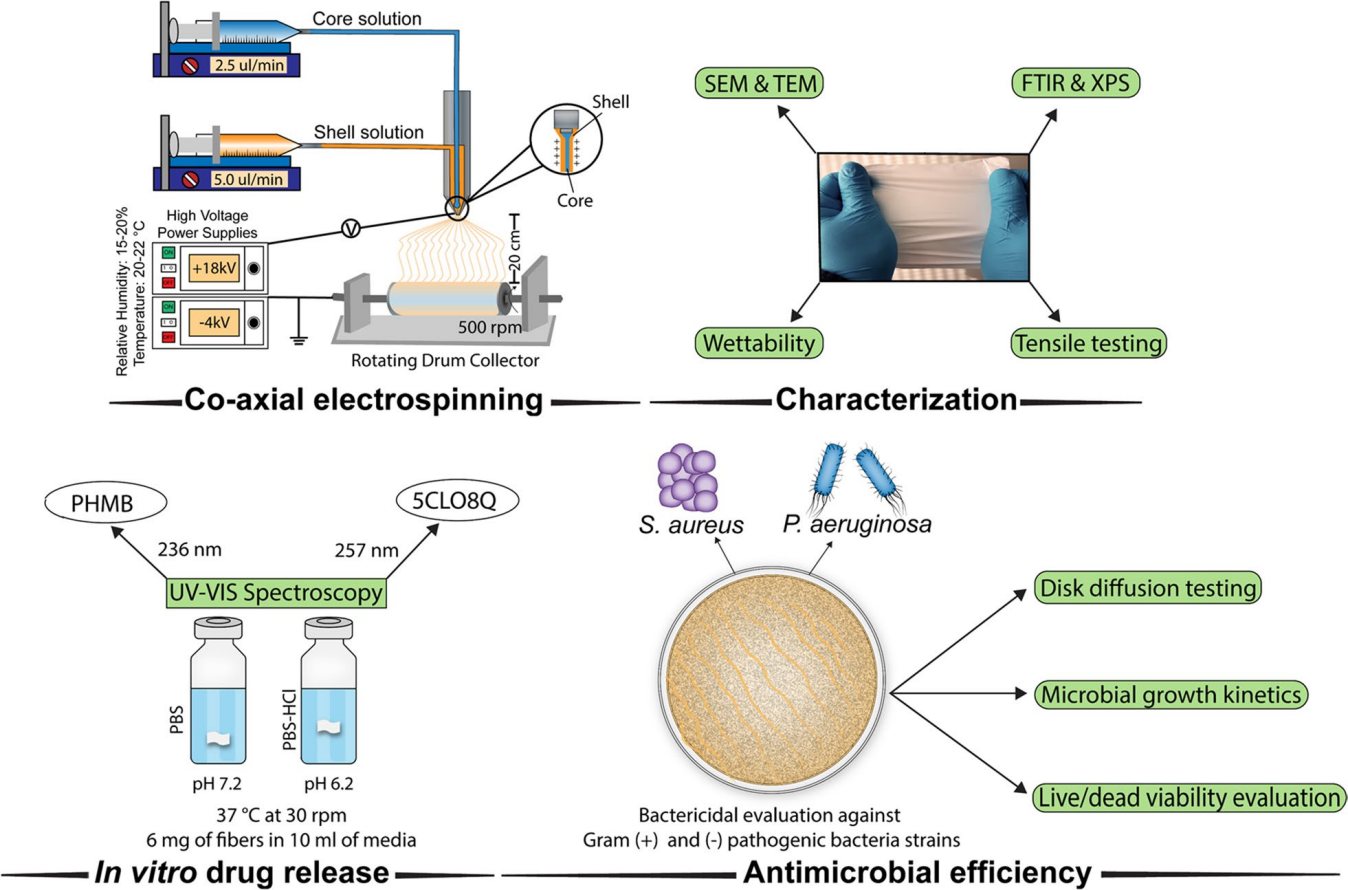
Schematic diagram depicting the fabrication methodology used to produce the core/shell PA6-PHMB/CS-5CLO8Q nanofibrous mats and the subsequent experimental and investigative scheme followed in this study

In this work, single CS-5CLO8Q and PA6-PHMB electrospun nanofibers (NFs) were produced and compared towards their mechanics and antimicrobial responses, to the composite core/shell structure, PA6-PHMB/CS-5CLO8Q. Polyethylene oxide (PEO) was added to the chitosan blends as a carrier polymer, to stabilize and improve the electrospinnability for homogenous fiber formation. The main goal of this study was the development of a construct that incorporates a dual drug release system of antimicrobial substances while combining the mechanical stability of PA6 with the cytocompatibility of CS. To get further insights into the parameters affecting the release of the drugs, the electrospun membranes were assessed morphologically and chemically. Drug release kinetic experiments were performed in vitro. While taking into account two of the most commonly associated bacteria species linked to surgical site infection of hernia meshes, Gram ( +) *Staphylococcus aureus* (*S. aureus*) and Gram (–) *Pseudomonas aeruginosa* (*P. aeruginosa*) were assessed in this study. The antimicrobial efficiency of the electrospun membranes was then evaluated via inhibition zone measurements, growth kinetics, live/dead staining, and visualized using SEM imaging (Fig. 1).

## Results and discussion

### Electrospun fiber morphology and core/shell structure

**Fig. 2:**
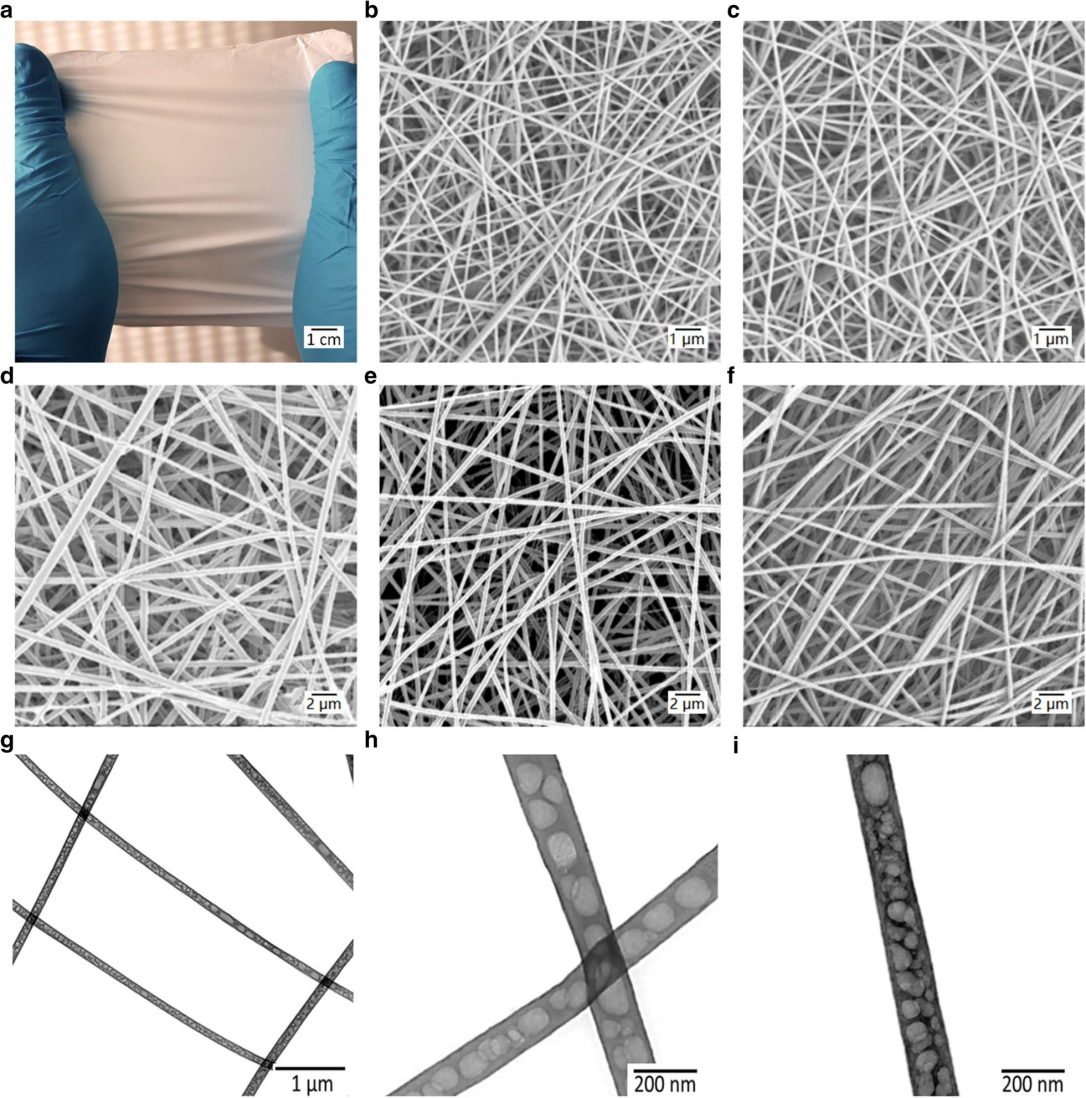
Nanofiber structure and surface morphology of the electrospun mats. **a** Macrograph of the electrospun mat. SEM micrographs of (**b**) CS, **c** PA6, **d** CS-5CLO8Q, **e** PA6-PHMB and **f** core/shell PA6-PHMB/CS-5CLO8Q. (**g**–**i**) TEM micrographs of the core/shell PA6-PHMB/ CS-5CLO8Q nanofibrous mats. (Core to shell feed rate: 2.5 and 5.0 µL min^−1^)

All electrospinning experiments were optimized towards their electrospinning parameters (humidity, temperature, flow rate, distance between the tip of the needle and the collector, potential difference, and needle diameter), as well as by tailoring the solution parameters (blend ratio, molecular weight, polymer concentration, viscosity, solvent system selection and compatibility) via parametric studies. The electrospinning system produced jets of single and core/shell fibers out of a stable Taylor cone in a continuous and homogenous manner. The morphology and corresponding fiber diameter distribution plots of the electrospun NFs are shown in the micrographs in Fig. 2 and Additional file 1: S3. To improve the homogeneity and spinning throughput of the CS NFs during the electrospinning process, 80:20 w/w ratio of CS: Poly (ethylene oxide) (PEO) was added directly to the dissolved CS polymer solution in all the experiments [37]. As CS is a polycationic polymer, in an aqueous solution, the electrospinnability of the polymer solution becomes quickly unstable due to polyelectric effects [38]. Numerous studies have shown that the repulsive forces between the ionic groups of the chitosan backbone augment during the electrospinning process affecting the homogeneous production of fibers [39]. By the addition of PEO as a backbone carrier polymer, the repulsion of the CS chains gets reduced due to the formation of H-bonds between the –OH of the PEO and the water molecules within the aqueous solvent system by acting as a proton acceptor [38]. This ultimately reduces the polyelectric effect and the degree of backbone chain entanglement, allowing for continuous and stable fiber production.

The SEM analysis (Fig. 2) demonstrates that all the fibers produced showed a smooth morphology, with no beads or major secondary artifacts present and a randomized fiber-matrix architecture. For all groups, fibers appeared to be uniformly distributed. All scaffolds revealed randomly oriented fibers collected on a rotating collector at a low speed (500 rpm).

As illustrated in Fig. 2b, homogenous bead-free CS NFs with an average fiber diameter of 101 ± 12 nm were successfully developed. PA6-only NFs (Fig. 2c) presented the thinnest smallest fibers, with an average fiber diameter of 88 ± 11 nm. Interestingly, CS NFs containing 15% w/w 5CLO8Q (Fig. 2d) with a fiber thickness ranging at 210 ± 31 nm were twice the diameter of the drug-free CS NFs for the same electrospinning parameters. As the addition of 5CLO8Q in the solution further upsurges the polycationic charges of the backbone chitosan chain, it can additionally influence the Ohmic flow and concurrently the convective flow, thus affecting its electrospinning behavior. Similarly, PA6 NFs containing 0.3% w/v PHMB (Fig. 2e) had an average diameter of 181 ± 22 nm, twice as thick as the drug-free PA6 NFs. The thickness of the fibers is generally influenced by the electrical conductivity of the polymer solution; by adding the antimicrobial substances, the electrical conducity increased, thus producing thicker fibers [40].

The drug-containing core/shell PA6-PHMB/CS-5CLO8Q NFs (Fig. 2f) had a slightly increased average fiber diameter compared to their counter-polymers, at 270 ± 68 nm. Compared to single polymer solutions, co-axial electrospinning increases the overall solution concentration (of the two separate polymer solutions) on the Taylor cone and has been shown to increase the overall fiber diameter [39]. The porosity was approximately 80–90% for all of the produced nanofibrous scaffolds. The morphological properties of the electrospun mats are summarized in Table 1.

**Table 1:** The morphological properties of the single and core/shell electrospun mats

Nanofibrous Scaffold	Feed rate (μL min^−1^)	Mean diameter (nm)	Density (Ints.μm^−2^)	Porosity (%)
PA6-PHMB	8.0	181 ± 22	4.05 ± 1.5	90 ± 3.2
CS-5CLO8Q	8.0	210 ± 31	1.83 ± 0.6	80 ± 4.5
PA6-PHMB/CS-5CLO8Q*	2.5/5.0	270 ± 68	1.56 ± 0.5	80 ± 4.4
Nylon-6 (PA6)	8.0	88 ± 11	1.54 ± 0.6	90 ± 1.8
Chitosan (CS)	8.0	101 ± 12	1.08 ± 0.3	91 ± 2.3

Electrospinning was carried out at 22 kV potential (+ 18/− 4 kV), from a needle tip-to-collector distance of 20 cm, using a rotating collector at 500 rpm *Core/shell nanofibers

The TEM analysis of the core/shell NFs was conducted by depositing electrospun fibers directly on TEM grids in a thin layer. The obtained micrographs can be seen in Fig. 2(g–i). A distinct morphology was observed, where a continuous phase of CS was apparent in the shell of the fibers, with a discontinuous PA6-PHMB phase presenting unique, consistent and well-distributed PA6 particles along the core of the fibers, possibly due to differences between the electric field-induced phase separation and differences in surface tension of the two materials. Kinetic factors relating to the rapid solvent evaporation due to differences between the solvent-systems of the core and shell polymer solutions may have played key roles in the formation of this structure [41, 42]. Polymer solutions with a concentration lower than the corresponding polymer entanglement concentration (ϕe), have been previously shown to yield electrospun NFs with beads-on-a-string configuration due to the jet’s increased volatility, relating to Rayleigh instabilities [43]. This morphology may indicate that the core-material was encapsulated independently and phase-separated from the shell-material's matrix wall. To further evaluate this unique structure, core/shell NFs were also produced with different flow rates under the same electrospinning parameters, but no differences were observed towards the morphological configuration of the core (Additional file 1: Figure S4).

Water contact angle (WCA) measurements were determined to evaluate the wettability properties of the core/shell NFs, an important parameter towards its drug release kinetics and antimicrobial behavior. The results presented in Fig. 2a indicate that the CS NFs containing 5CLO8Q presented a WCA of 66° ± 9°, compared to 75° ± 5° for the drug-free CS NFs. As expected, the addition of PHMB in the PA6 NFs increased the hydrophobicity of the produced scaffolds due to the inherent hydrophobicity of the pure drug. The core/shell NFs presented the lowest contact angle of 50° ± 8°, which indicates that the composite presents improved wettability properties. Similarities of the core/shell and CS-5CLO8Q NFs towards their pore area and the better pore distribution of core/shell NFs may have played key roles in further improving the wettability of the composite structure's surface. Several studies have shown that the surfaces of more hydrophilic NFs encourage better eukaryotic cellular attachment behavior and improved proliferation [44]. Hydrophilic surfaces have been proven to provide more suitable binding sites for cellular adhesion, leading to more efficient scaffold colonization, thus making the material increasingly cell compatible [45]. Interestingly, the adhesion of bacterial cells on hydrophilic material is influenced by the surface energy of the media (peritoneal fluid, blood, etc.) in which the bacteria are suspended, which is however, substantially inferior to the bacterial surface energy [46]. In general, bacteria attach widely on moderately hydrophobic surfaces that carry lower surface energy [46]. Furthermore, increasingly hydrophilic substrates present low zeta potential values, which limit bacterial binding by inducing repulsive interactions [47].

### Chemical composition of the core/shell electrospun NFs

**Fig. 3:**
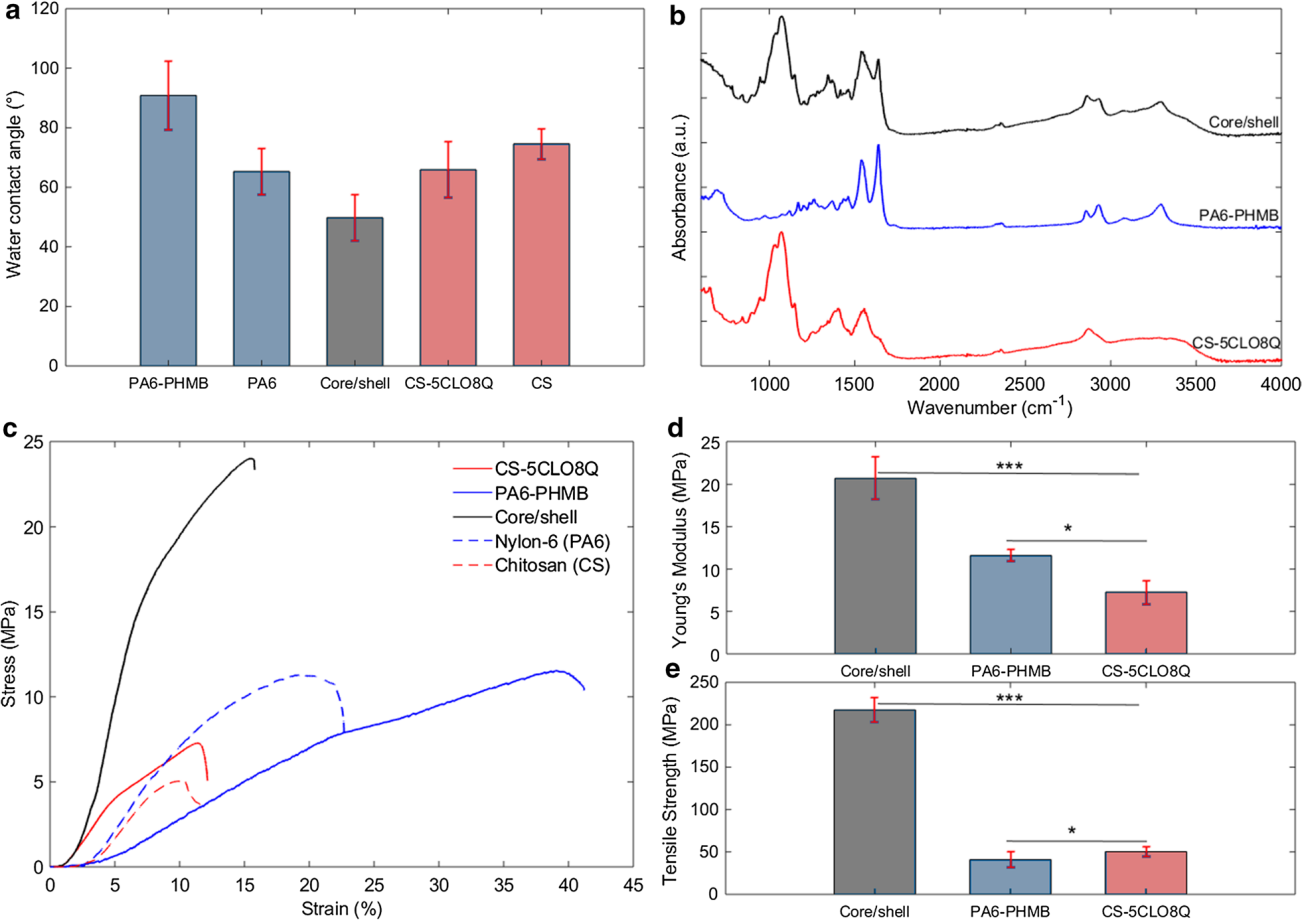
Electrospun materials properties. **a** Water contact angles of the electrospun NFs with and without the antimicrobial agents and the drug-containing core/shell NFs. **b** FTIR-ATR spectra, **c** Representative tensile stress–strain curves of the drug-containing, drug-free, and core/shell electrospun mats. **d** Young's modulus and **e** Ultimate tensile strength of the drug-containing and core/shell NFs. Error bars = standard deviations, where star symbols indicate significant difference p-values, *** p < 0.001 and * p < 0.05. *Core/shell is PA6-PHMB/CS-5CLO8Q

FTIR spectroscopy was performed to characterize the functional groups present on the CS-5CLO8Q, PA6-PHMB, and core/shell nanofibrous mats (Fig. 3b, Additional file 1: Figure S5). The IR spectrum of the CS-5CLO8Q fibers indicates the presence of a broad band between 3150–3500 cm^−1^, characteristic of –OH stretching, and –NH stretches of primary amino groups (Amine I). Possibly, intermolecular –OH and –CH_2_OH hydrogen bond interactions between 5CLO8Q and CS could contribute to the band broadness, observed in the region corresponding to 3380–3420 cm^−1^ [48]. The peak observed at 2867 cm^−1^ can be attributed to –CH stretching, while the peak at 1557 cm^−1^ may correspond to the amide II C–N stretching coupled with –NH in-plane deformation [49]. The sharp bands present around 1033 cm^−1^ may refer to the –C–O–C stretching in the CS polymer [48]. Based on the region-specific spectral scans (2000–1200 cm^−1^), the amide I characteristic C = O stretching of the N-acetyl group appears at 1651 cm^−1^ (Additional file 1: Figure S6). The peaks at 1414 cm^−1^ and 1367 cm^−1^ may correspond to the bending of –CH_2_ and –OH, respectively.

The PA6-PHMB spectrum presents a strong band at 1639 cm^−1^ that corresponds to the C = O stretching, while the peak at 1542 cm^−1^ clearly corresponds to the characteristic in-plane N–H bending of amide groups. The weak trans amide conformation between 1310–1350 cm^−1^ and 1440–1490 cm^−1^ indicates that the amide region is in a gauche-conformation [50, 51]. The two medium peaks at 2930 and 2855 cm^−1^ represent C–H stretches of the polyamide backbone. In addition, the peak at 1263 cm^−1^ may be attributed to the amide C–N stretch.

The spectrum of the core/shell PA6-PHMB/CS-5CLO8Q NFs confirms the presence of both polymers in the final composite structure. The characteristic peaks of the two polymers were present in the core/shell structure's spectrum. The –NH stretching appears to have been slightly broadened and less intense at 3292 cm^−1^, which could be due to hydrogen bond interactions between the two polymers. The C=O and –CH stretch of the Polyamide appeared to stay stable at 1638 and 1542 cm^−1^, respectively, but at a lower intensity. All of the chitosan specific area spectra appear to be present at the core/shell nanofibrous mats at lower intensities. No new peaks were observed on the composite core/shell structure.

### Surface chemistry of the core/shell PA6/CS NFs

The XPS analysis, incorporating an information depth of around 10 nm, was conducted to compare and understand the surface chemistry of the electrospun NFs and to support the measurements obtained by TEM. The survey scans for CS-5CLO8Q, PA6-PHMB, as well as the core/shell NFs produced, are presented in Table 2 (Additional file 1: Figure S7). The PA6-PHMB and CS-5CLO8Q NFs were evaluated based on theoretical values. Effectively, the core/shell fibers appear to carry similar values to that of the CS, with a high atomic percent content of O1s and lower N1s as opposed to the PA6-PHMB fibers. Further, chlorine (Cl 2p), a selective element for the antimicrobial 5CLO8Q was not apparent in the CS-5CLO8Q nor in the core/shell fibers, indicating entrapment of the antimicrobial substance towards the core of CS (XPS exhibits an information depth of 10 nm). The 1.3% increase in the amount of nitrogen present in the core/shell composite, compared to that of CS-5CLO8Q, could denote small irregularities at the fiber surfaces due to the stabilization period of the Taylor cone during the co-axial electrospinning process. Further on, the characteristic component peaks for each nanofibrous scaffold appeared to follow the same logic – where the core/shell fibers showed similar patterns to the CS NFs (Additional file 1: Table S1).

**Table 2:** Surface chemical composition of the electrospun NFs

Fibers	C1s		N1s		O1s	
	eV	Atom%	eV	Atom%	eV	Atom%
CS-5CLO8Q	284.4	65.4	399.2	3.0	530.4	31.6
		(61)		(5)		(34)
PA6-PHMB	284.5	75.5	397.8	12.2	531.3	12.3
		(75)		(13)		(12)
Core/shell*	284.5	65.8	398.1	4.2	530.8	30.0
CS-only	284.5	72.3	399.1	2.1	530.8	25.6
PA6-only	284.4	77.0	399.0	11.3	532.3	11.9

The percentages in brackets () refer to the theoretical values of each polymer *Core/shell is PA6-PHMB/CS-5CLO8Q

### Mechanical characteristics of the produced core/shell fibers

The mechanical properties of the CS-5CLO8Q, PA6-PHMB, and core/shell fibers are shown in Fig. 3(c–e) and Table 3. As can be seen by the representative stress/strain curves in Fig. 3c, the core/shell NFs successfully improved the mechanical stability of CS by the addition of the PA6 in the core of the fibers.

**Table 3:** Overview of the mechanical properties of the electrospun scaffolds

Nanofibrous scaffold	Ultimate tensile strength (MPa)	Elongation at break (%)	Young's modulus (MPa)
Chitosan (CS)	6.6 ± 0.8	12.5 ± 4.1	50.5 ± 8.3
Nylon-6 (PA6)	10.7 ± 0.6	20.3 ± 2.8	64.9 ± 7.8
CS-5CLO8Q	7.3 ± 1.4	10.4 ± 1.4	50.3 ± 5.4
PA6-PHMB	11.6 ± 0.7	36.3 ± 7.5	41.0 ± 8.8
Core/shell*	20.7 ± 2.5	14.1 ± 2.1	217.5 ± 12.6

^*^Core/shell is PA6-PHMB/CS-5CLO8Q

The CS-5CLO8Q and PA6-PHMB non-woven electrospun mats presented an ultimate tensile strength (UTS) of 7.3 ± 1.4 MPa and 11.6 ± 0.7 MPa, respectively. The UTS of the drug-free electrospun mats falls into the same range as the drug-containing NFs, while the UTS of the thicker in diameter core/shell NFs outperformed the single polymer fibers at 20.7 ± 2.5 MPa. As expected, PA6-PHMB presented the highest tensile strain at 36.3 ± 7.5%, while the PA6-only NFs appeared to be stiffer with an elongation at break at 20.3 ± 2.8%, probably owing to fiber-to-fiber interaction variations due to differences in the fiber diameters among the two groups. The core/shell fibers seemed to be influenced by the presence of CS and were found to be stiffer than the PA6-PHMB NFs with an elongation-at-break at 14.1 ± 2.1%, which was nevertheless an improvement from 10.4 ± 1.4% for the CS-5CLO8Q electrospun mats. The Young's modulus of the composite core/shell fibers imposed a four-fold increase to 217.5 ± 12.6 MPa, compared to 50.3 ± 5.4 MPa and 41.0 ± 8.8 MPa, for the CS-5CLO8Q and PA6-PHMB, respectively.

The mechanical properties of the electrospun PA6-containing NFs are in agreement with previously published work [52]. However, PA6 nanofibrous mats produced via electrospinning are inferior to those produced by microfiber fabrication techniques, such as melt spinning, which could possibly be explained due to the lower degree of chain orientation of the asymmetrically electrospun (as-spun) NFs [53, 54]. Nonetheless, further investigations of the single fibers and fiber-to-fiber friction properties could provide more insights. The relatively low mechanical properties of the as-spun CS-containing non-woven mats, concur with previous stress/strain studies based on NFs produced from CS blends incorporating PEO [54, 55]. Interestingly, the core/shell NFs exceeded the CS and PA6 tensile properties. Composite NFs present higher tensile strength compared to their counterparts due to an improved molecular orientation and conformation, crystallinity, chemical interactions, etc. The produced core/shell fibers had an increased fiber diameter, compared to the pure fibers—a factor which effectively plays a role in this improved mechanical stability of the composite structure. Additionally, energy dissipation due to the phase-separated "beads-in-fiber" morphology, may have also played a role in influencing the toughness of the composite fibers [43]. Such interactions can possibly allow for a better distribution of the energy crossing through the PA6 containing core, stabilizing the CS-containing sheath, thus retarding its fracture.

### Drug release mechanism of the antimicrobial NFs

Predominately, electrospun NFs produced via the blending of a polymer with a drug typically display a rapid burst into the release medium due to small diffusion pathways, which can be influenced by the drug to polymer affinity and the solubility of the drug in the release medium [56]. On the other side, co-axially electrospun drug-loaded systems allow for the encapsulation of an antimicrobial agent within the core of the fibers, increasing the diffusion pathway and thus, retarding the initial burst and sustaining a more modulated release. The release kinetics were examined at pH 7.2 and 6.2 to resemble a physiological and a slightly more acidic environment that could exist in an intra-abdominal diseased bacterial infected tissue [57], respectively.

#### The release kinetics of the single-drug containing NFs

**Fig. 4:**
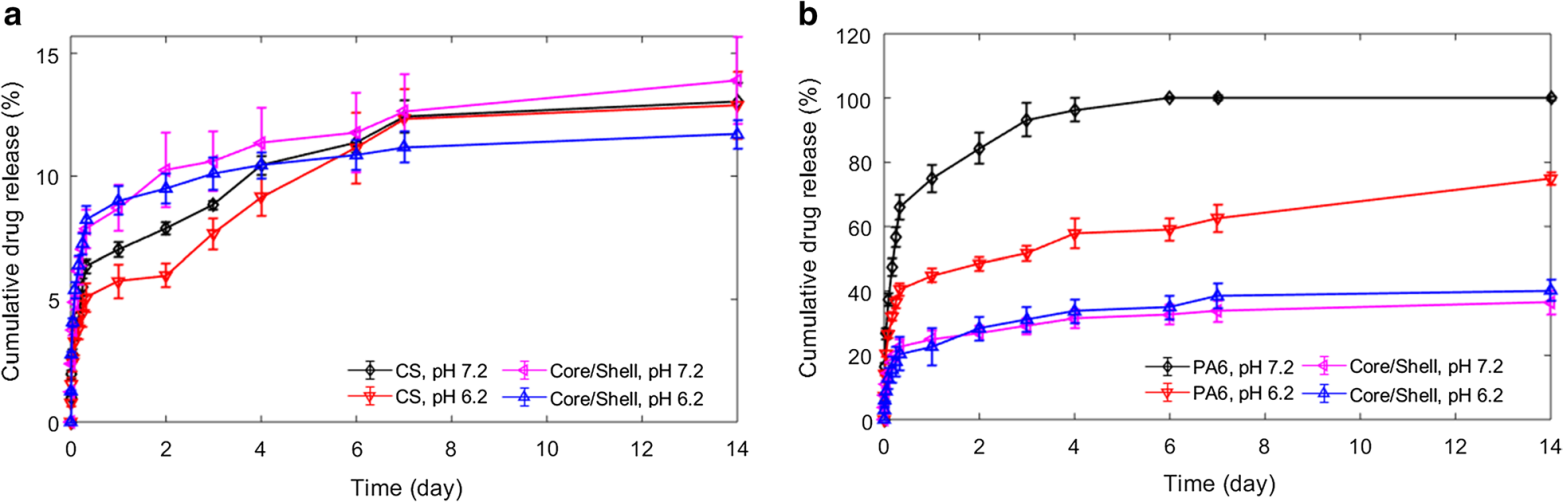
Comparative cumulative release of (**a**) 5CLO8Q and (**b**) PHMB, from the antimicrobial CS and core/shell NFs, at pH 7.2 and 6.2. Error bars = standard deviations. n = 6, deriving from two independent drug release studies conducted using fiber mats from different electrospinning batches. *Core/shell is PA6-PHMB/CS-5CLO8Q

For the CS NFs containing 5CLO8Q, an initial burst of 7.1 ± 0.68% for pH 6.2 and 6.3 ± 0.27% for pH 7.2 was apparent within the first 6 h of incubation, followed by a slow release that did not exceed 15% of the loading capacity of the NFs in a period of 14 days (Fig. 4a). Quinolines are poorly water-soluble and, thus, strongly affected by the release media. The release of 5CLO8Q from chitosan appeared to follow a non-Fickian release (n = 0.81 at pH 7.2 and 0.85 at pH 6.2) based on the Korsmeyer–Peppas model, which indicates that CS erosion, swelling and dissolution rate, are critical in the release of 5CLO8Q. Further, entrapment of the molecule towards the core of the CS fibers could also rationalize this phenomenon as the XPS results did not present the corresponding chlorine, present in the benzene ring of the 5CLO8Q molecule at the surface of the NFs, as well as the hydrogen bonding interactions between CS and 5CLO8Q presented by FTIR. The same pattern, as expected, was observed for the 5CLO8Q present in the core/shell NFs, as the antimicrobial agent was present within the CS-sheath. The 60 nm fiber diameter difference between the CS and core/shell NFs did not appear to affect the release profile of the substance.

In the PA6 NFs containing PHMB, the release of the substance appeared to be strongly affected by the pH of the medium (Fig. 4b). At pH 7.2, a burst release corresponding to 56.7 ± 3.20% in 6 h was observed, where 100% of the PHMB contained by the fibers was released within 4 days. Nonetheless, at pH 6.2, an initial release of 30.6 ± 2.70 in 6 h was apparent, with 63.5 ± 1.20% of the contained drug being released within a 14-day period. The release from PA6 fibers followed a Fickian diffusion behavior (n = 0.26 at pH 7 and 0.40 at pH 6.2). The cationic nature of the very basic biguanide molecules makes PHMB gradually positively charged as the pH decreases. In an aqueous environment, the PHMB conformation alternates with the hydrophobic methylenic region facing inwards and the biguanide groups facing outwards [58]. Polyamides have been shown to be significantly affected by pH, where the isoelectric point present at pH 7.0 shifts as the pH acidifies due to pH-dependent protonation, which ultimately causes swelling, as the pH gets greater than the pKa of the polymer [59, 60]. That increase in the net charge of the biguanides could ultimately shift the isoelectric equilibrium present at pH 7.0 and thus, become more susceptible to chemical interactions between the two molecules, retarding its release at pH 6.0.

#### The effect of the core/shell structure in the release of PHMB

The core/shell NFs appear to follow a release pattern, for PHMB present in the core, not influenced by the pH (Fig. 4b). Firstly, a small burst release accounting to approximately 20.3 ± 2.2% at pH 7.2 and 17.9 ± 4.6% at pH 6.2 of the encapsulated PHMB within 6 h was apparent. This could be due to a proportion of the electrospun drug appearing near or at the surface of the core/shell fibers [61], as well as possible inconsistencies at the beginning of the electrospinning process. Afterwards, a steady release was evident at a similar rate for pH 6.2 and 7.2 for the period of 14 days, with a maximal release of 36.45 ± 3.5% at pH 7.2 and 40.2 ± 3.4% at pH 6.2. Noteworthy, the core/shell NFs appeared to follow a non-Fickian release mechanism (n = 0.85 for pH 7 and pH 6), which confirms that the chitosan present in the sheath governed the release of the PHMB in the core/shell NFs. The positively charged CS permits the absorption of water molecules and simultaneously the diffusion of PHMB to the release medium. Under this model and conditions, and while considering CS undergoing consistent degradation, we can contemplate a complete theoretical release of PHMB in the media within a period of 45 ± 5 days at pH 7.2 and 36 ± 4 days at pH 6.2.

### Antimicrobial activity of the single and core/shell NFs

**Fig. 5:**
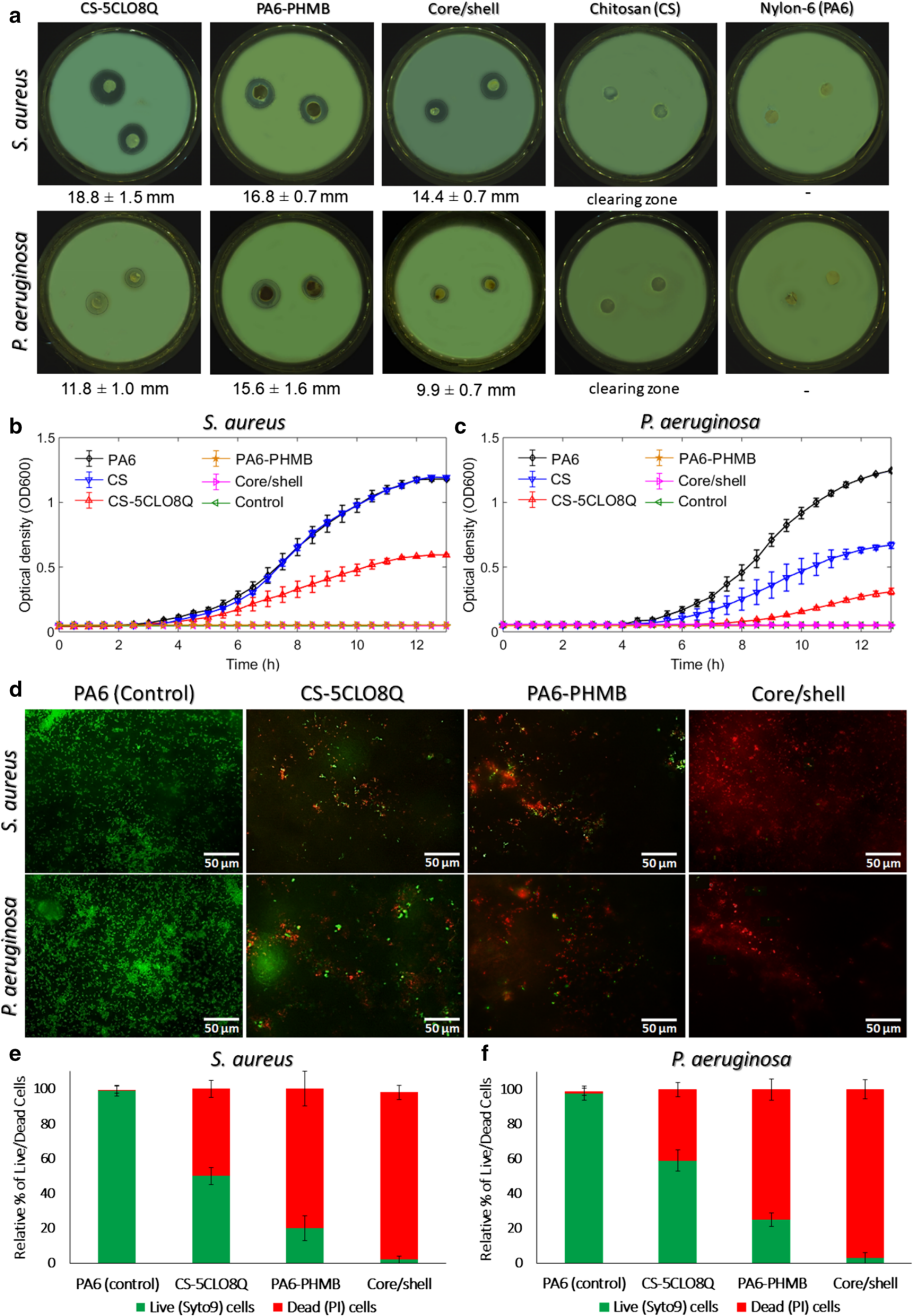
Antimicrobial properties of the electrospun mats. **a** Macrographs of the disc diffusion test with the corresponding radius of the zone of inhibition (mean ± SD). **b***S. aureus* and **c***P. aeruginosa* growth reflected as optical density (OD600) over time, of the electrospun CS, CS-5CLO8Q, PA6, PA6-PHMB, core/shell NFs and control. **d** Fluorescence microscopy assessment via live/dead BacLight Syto9/Propidium iodide (PI) bacterial viability assay, and (**e**–**f**) relative percentage of live (green) and dead (red) cells upon exposure to the variant groups of electrospun fibers, quantified from the fluorescent intenstity of the microscopic images. All data are shown as average values ± standard deviations from two independent experiments (n = 6). *core/shell is PA6-PHMB/CS-5CLO8Q

The antimicrobial effect of the electrospun NFs was investigated against *S. aureus* and *P. aeruginosa*, the two most frequently encountered pathogens associated with surgical site infection (SSI). The zones of growth inhibition shown in Fig. 5a indicate that no zone of inhibition was evident for the PA6-only nanofibrous mats, whereas a zone of clearing was present on the CS-only electrospun mats. Since the inhibitory potency was apparent around the borders of the CS-only disks, we can denote that no chitosan derivatives were secreted along the surrounding area.

The CS-5CLO8Q NFs showed the strongest inhibition zones against *S. aureus* with the PA6-PHMB fibers presenting an evident inhibition against *P. aeruginosa*, while the core/shell NFs appeared to be effective against inhibiting both bacterial strains. The polymer matrix material in which the biocide agent is incorporated largely impacts the electrospun mat's degradation [62]. The zones of inhibition of the composite core/shell fibers cannot be directly compared with the single polymer-drug systems examined, as the degradation rate will differ based on the polymer system's composition and corresponding fiber properties.

A small fraction of studies that investigate the effect of electrospun antimicrobial fibers has used *P. aeruginosa*, as it is much less susceptible to antibiotics and antimicrobial agents [63] in comparison with *Escherichia coli*, which is commonly used as the model Gram-negative representative organism in the majority of the published work. Different responses among the two strains are anticipated as they differ greatly towards their structural morphology, shape, and metabolic responses. The inhibition zones indicated that the produced antimicrobial scaffold showed a less robust response against *P. aeruginosa* but effectively presented clear zones of inhibition, even if less intense.

To further evaluate and quantify the bactericidal responses of the electrospun mats, 12 h growth kinetic studies (Fig. 5b; *S. aureus*, Fig. 5c; *P. aeruginosa*) were performed for each bacterial strain after being exposed on the electrospun membranes. *S. aureus* appears to be partially susceptible to the CS-5CLO8Q NFs, with a retarded growth time and lower optical density in comparison with the PA6-only and CS-only electrospun mats. Interestingly, the PA6-PHMB and core/shell NFs effectively suppressed the growth of the bacteria upon exposure to the antimicrobial NFs. The more resilient *P. aeruginosa* strain was not found to be affected by the presence of the CS-only NFs, where the CS-5CLO8Q NFs showed a reduced optical density as opposed to the CS-only and PA6-only electrospun mats. The PHMB-containing core/shell and PA6-PHMB NFs eliminated the growth of the Gram-negative bacteria upon exposure.

The bacterial kinetics experiments contradicted the inhibition zone observations, where 5CLO8Q appears to be the most effective in inhibiting the growth of the bacteria examined. This could be due to the CS-5CLO8Q electrospun mats being capable of secreting the antimicrobial substance on the solid-agar plates repelling bacterial growth in the surrounding areas, but nonetheless, being ineffective in preventing the growth of the bacteria when placed at the surface of the nanofibrous matrices. These results indicate the strong bactericidal activity presented by the PHMB, even at low concentrations (0.3% w/v). The composite core/shell NFs appeared to completely repel the growth of these two bacterial strains commonly associated with SSI, thus successfully presenting an antiadhesive-like surface, with the biocompatibility properties offered by the presence of a naturally derived CS and the synergic antimicrobial properties of these two substances.

**Fig. 6:**
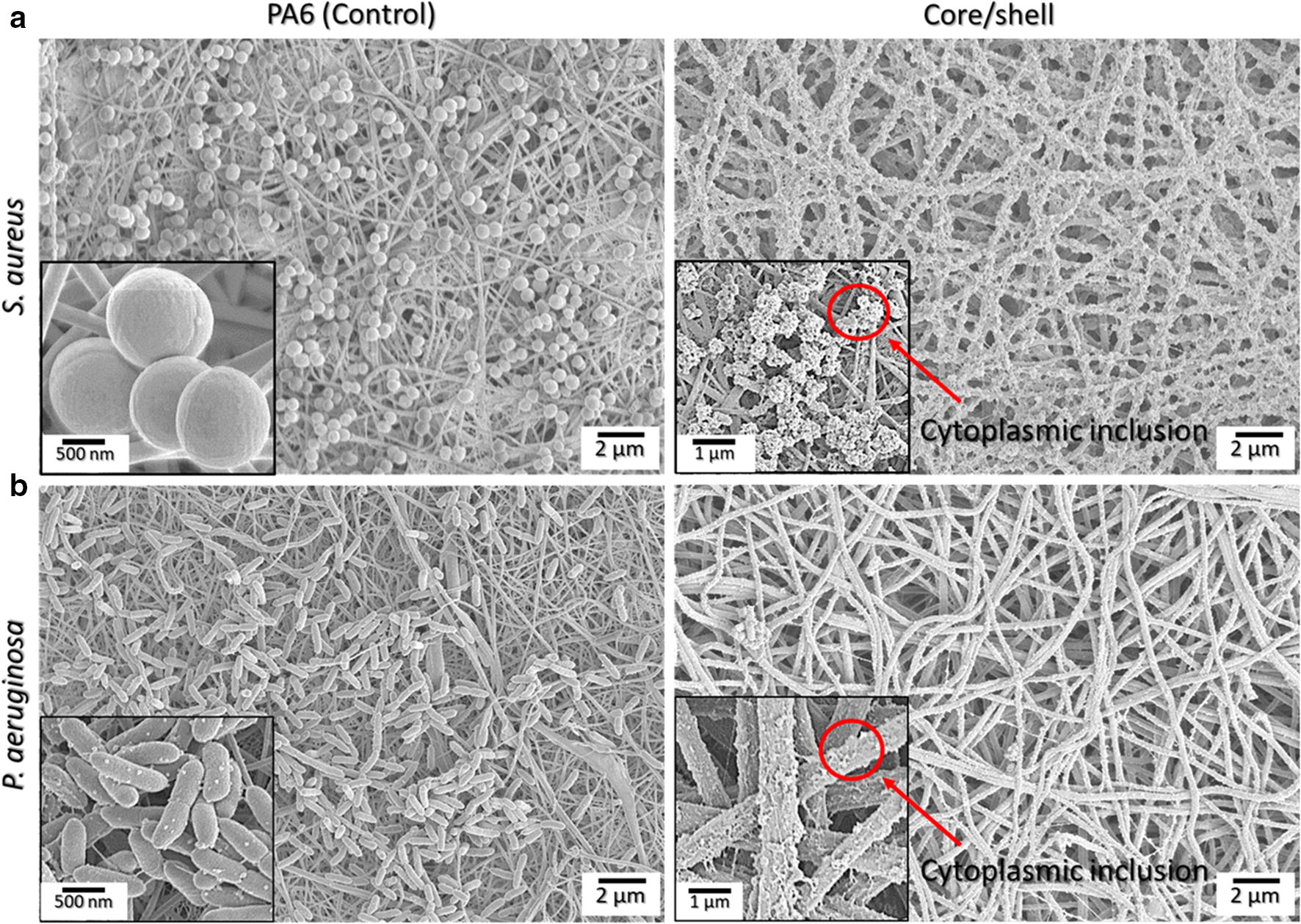
SEM micrographs of (**a**) *S. aureus* and (**b**) *P. aeruginosa* morphological properties upon exposure for 6 h to the electrospun core/shell and PA6 (control) nanofibrous mats. Red arrows indicate cytoplasmic inclusions of dead or membrane damaged cells

The susceptibility of *S. aureus* and *P. aeruginosa* to the core/shell NFs was further evaluated via live/dead staining fluorescence microscopy and by investigating the bacteria morphological characteristics via SEM microscopy. Syto9 (green) penetrates bacterial membranes and binds to the nucleic acid of both live and dead bacteria, while PI is impermeable to intact membranes and can only penetrate damaged bacteria binding on nucleic acid material. As can be observed in Fig. 5d, upon exposure to the antimicrobial NFs, the core/shell mats elicited the most potent bactericidal efficiency with the majority of the dye binding to damaged membrane cytoplasmic nucleic acids. Figure 5(e, f) indicates the relative percentage of live/dead bacteria upon exposure to the variant electrospun mats as quantified by the fluorescent images, where it is clearly apparent that exposure to the core/shell structure is the most effective in decimating the growth of the two bacterial strains examined. Further on, the SEM micrographs presented in Fig. 6(a, b) compare the PA6-only with the core/shell antimicrobial NFs indicating the distinct morphological differences of healthy and dead bacteria upon exposure to the electrospun mats. The normal cocci and rods morphology of *S. aureus* and *P. aeruginosa*, respectively, on the PA6 NFs surface, can be visibly different to that of the characteristic cytoplasmic inclusion, which does not convey any metabolic activity, of dead bacteria matter present upon exposure to the antimicrobial core/shell electrospun mats.

## Conclusion

In summary, a novel nanofiber-based antimicrobial system was successfully developed through the tailoring of the materials combined, fiber structure, and electrospinning parameters, leading to core/shell PA6-PHMB/CS-5CLO8Q NFs. The core/shell electrospun mats presented homogenous morphology, with a smooth surface. A consistent "beads-in-fiber" architecture was observed along the core of the core/shell NFs. This unique architecture gave the PA6 present in the core the right molecular configuration, and to the CS present in the shell the appropriate fiber morphology, to provide enhanced mechanical strength; an essential feature for the development of a hernia mesh implant. In vitro evaluation of the antimicrobial activity of the electrospun meshes via agar disk-diffusion testing, dynamic growth kinetics, live/dead staining and SEM microscopy illustrated a compelling, efficient bactericidal activity against *S. aureus* and *P. aeruginosa*. The binary antimicrobial system presented spatial controlled release, which followed a non-Fickian release model directed by the CS present in the shell of the structure. This study would like to advert the profound antimicrobial properties of 5CLO8Q and PHMB, and their respective possibilities for encapsulation to tailor drug release kinetics. Conclusively, these findings advocate the great potential behind the co-axial electrospinning fabrication technique for the development of advanced drug-delivery systems based on NFs. This electrospun material can ultimately find applicability in the prevention of bacterial infections of surgical mesh surfaces.

## Experimental

### Materials

Chitosan powder (degree of de-acetylation 85/100) was purchased from Heppe Medical Chitosan GmbH, Germany. Nylon-6 (Polyamide-6; PA6) pellets, Poly(ethylene oxide) (PEO, Mw 900,000 g mol^−1^), 5-Chloro-8-quinolinol (5CLO8Q, Mw 179.60 g mol^−1^), Poly(hexamethylene biguanide) hydrochloride (PHMB, Mw 213.33 g mol^−1^), Ruthenium(III) chloride (RuCl_3_, Mw 207.43 g mol^−1^) and sodium hypochlorite (NaClO, 74.439 g mol^−1^) were purchased from Sigma-Aldrich, USA. The solvents formic acid (FA, purity ≥ 95%) and acetic acid (AcOH, purity ≥ 99.8%) were acquired from Sigma-Aldrich, USA. Phosphate-buffered saline (PBS, pH 7.4), Mueller–Hinton broth (MH), Brain–heart infusion (BHI), Lysogeny broth (LB) and agar were purchased from Sigma-Aldrich, USA. Baclight live/dead bacterial viability kit was purchased from Thermo Fisher Scientific, USA. *Staphylococcus aureus* (ATCC 6538) and *Pseudomonas aeruginosa* (ATCC 43,390) were purchased from the American Type Culture Collection, USA.

### Solution preparation and electrospinning

Master solutions were prepared by dissolving 3% w/v total concentration of Chitosan/PEO 80/20 w/w ratio in 50% aqueous acetic acid (referred to as, CS) and 21% w/v PA6 in formic acid. Then, 5CLO8Q 15% w/w of the total polymer solution and 0.3% w/v of PHMB was added to the CS and PA6 solutions, respectively. The solutions were placed on a shaker at 200 rpm overnight at room temperature.

CS and PA6 solutions were electrospun using a horizontal electrospinning setup with a coaxial feeding nozzle of 0.9 mm diameter for the shell and 0.57 mm diameter for the core, where fibers were deposited on baking paper on a rotating collector at 500 rpm. The solutions were fed into the core–shell needle, via two separate automatic pumps. The flow-rate was set at 2.5 µL min^−1^ for the core and 5 µL min^−1^ for the shell solution. Single CS-5CLO8Q and PA6-PHMB, as well as CS and PA6 solutions, were also electrospun using a 0.58 mm needle (8 µL min^−1^ flow rate). For all the experiments conducted, the distance between the tip of the nozzle and the surface of the collector was set at 20 cm, and electrospinning was conducted under a potential difference of 22 kV (+ 18 kV needle, − 4 kV collector) using a DC power supply at 0.1 mA current. The relative humidity was kept between 15–20%, whereas the temperature was kept between 20–22 °C using a climatic chamber. In order to produce uniform nonwoven electrospun mats, each experiment ran for 4–6 h.

### Fiber morphology

Scanning electron microscopy (SEM, Hitachi S-4800, Hitachi-High Technologies, Japan) was used to appraise the fiber diameter, as well as the porosity and the inner surface morphology of the electrospun mats. The specimens were sputter-coated with a layer of 8 nm gold to form a conductive surface and scanned at 2 kV. The mean fiber diameter was measured using the ImageJ software (National Institutes of Health, USA); where at least three different SEM micrographs were chosen, and randomly a total of 90–120 fiber width values were measured per sample. The fiber diameter, porosity and density of the nanofibrous scaffolds were then evaluated using the ImageJ add-on ‘DiameterJ’ from n ≥ 1000 fibers. The margin of error between the manually calculated and automatically measured computations was, in all cases ≤ 5%. Porosities were determined via threshold image analysis of the SEM micrographs using ImageJ. The density of the fibrous scaffolds was determined as ‘intersections per μm^2^, using DiameterJ, where: (the average length of the fibers) / (radius values) from four points for each fiber per image were analyzed. Then, the (Total number of intersections) / (specific areas, in pixels) × 10^4^ was calculated, which corresponds to 1/1000^th^ points per pixel of image.

Transmission electron microscopy (TEM, Zeiss EM 900, Carl Zeiss Microscopy GmbH, Germany) at 80 kV was utilized to confirm the core/shell structure of the electrospun fibers. Ethanol was used to mount the TEM copper grids to the surface of the collector, on which the fibers were directly deposited for a few seconds. The fiber-containing grids were stained by placing them on a desiccator containing a Ruthenium tetroxide (RuO_4_) solution at the gaseous phase, prepared by dissolving RuCl_3_ in 10 wt% NaClO solution [64], for 2 h, in order to attain an increased contrast between the two polymers when exposed to the transmission electron beam of the TEM microscope.

1 $$2\,{\text{RuCl}}_{{3}} \cdot 3\,{\text{H}}_{2} {\text{O}} + 8\,{\text{NaClO}} \to 2\,{\text{RuO}}_{4} + 8\,{\text{NaCl}} + 3\,{\text{Cl}}_{2} + 6\,{\text{H}}_{2} {\text{O}}$$

### Chemical characterization and wettability

Fourier-transform infrared spectroscopy (FTIR) was performed using an FTIR spectrophotometer (Varian 640-IR, Varian Medical Systems, USA). The measurements of each dried electrospun fiber mat were carried out using the ATR-crystal mode in mid-infrared scanning range of 4500–600 cm^−1^ with a spectral resolution of 2 cm^−1^. Each spectrum obtained was the average of 126 scans to enhance the signal-to-noise ratio.

The surface chemistry of the electrospun mats was analyzed by X-ray photoelectron spectroscopy (XPS) taken with a PHI 5000 VersaProbe II (USA) using an Al Kα X-ray source. The energy resolution of the spectrometer was set to 0.8 eV/step at a pass-energy of 187.85 eV for the survey scans. Carbon at 284.5 eV was used as reference to correct for charge effects. The elemental compositions were determined using instrument-dependent atom sensitivity factors. The photoelectron-transitions of C1s, O1s, and N1s were chosen to determine the surface elemental concentration of the fibers. The data were analyzed using the CasaXP software (Casa Software Ltd, UK).

The water contact angle (WCA) measurements of the electrospun membranes were obtained using a dynamic water drop machine (Drop Shape Analyzer DSA25, Krüss, Germany). For each scaffold, at least 8 measurements were obtained by dispensing a 5 µL droplet of ultrapure HPLC-grade water on the surface of the electrospun mats allowing for the droplet to settle for 10 s. The values were computed with the contact angle add-on of the ImageJ software, and the average values ± standard deviation are presented.

### Mechanical properties

For the mechanical characterization, uniaxial tensile testing was performed with a universal testing machine (Z100-Retroline, Zwick-Roell, Germany) using a 10 N load cell at a strain speed of 10 mm min^−1^. The dried electrospun membrane were placed into the climatized room under standard atmospheric conditions (22 ± 2 °C and 65 ± 5% RH) for 48 h prior to the measurements. The samples were cut into rectangular 10 × 40 mm strips; weighed, and the thickness was measured using a profilometer (XP-1 Stylus Profiler, Ambios Technology, USA). All samples weighed 1–2 mg and the thickness was between 0.05–0.15 mm. The formulas used for calculating the ultimate tensile strength (UTS) and extension at break were as follows:

2 $$Ultimate\,tensile\,strength \,\left(\text{MPa}\right)=\frac{Stress\, (\text{N})}{Speciment\;thickness \,\left(\text{mm}\right) \times Specimen \,width\,(\text{mm})}$$

3 $$Elongation\,at\,break\,(\%)=\frac{Specimen\;elongation\,(\text{mm})}{Original\;length\,(\text{mm})} \times 100$$

The Young's modulus (E) was determined from the slope of the linear segment of the stress/strain curve obtained for each specimen. At least six specimens were prepared for every type of electrospun mat, and the average values are represented as the mean ± SD.

### In vitro drug kinetics studies

The dried electrospun mats were cut into pieces weighing ~ 6 mg and placed in 20 mL centrifuge tubes containing 10 mL of PBS pH 7.2 or PBS-HCl pH 6.2. All the vials were incubated at 37 °C and shaken at 30 rpm. At specified time intervals, 1 mL of release medium was pipetted and replaced with equal volume of fresh solution. To evaluate the drug release profile of the single CS-5CLO8Q and PA6-PHMB, as well as that of the core/shell PA6-PHMB/CS-5CLO8Q NFs, Ultraviolet–visible (UV) spectroscopy (SynergyMX, BioTek Instruments, USA) was employed—based on the specific absorption spectra of each drug. For the 5CLO8Q in PBS medium a designated peak was ascertained as the average of 247/257 nm and for PHMB at 236 nm (Additional file 1: Figure S8). Fortunately, no secondary absorbance peaks at the corresponding wavenumbers were present between the two antimicrobial substances, and we could thus further assess the binary-drug core/shell NFs system. The cumulative release of the antimicrobial compounds from the electrospun fibers was expressed based on the standard calibration curve of each substance for the corresponding medium (Additional file 1: Figure S9). All measurements were taken in quadruplicates, and the results are expressed as the mean ± standard deviation (SD) of two independent electrospun mats from different batches.

To further investigate the drug release mechanisms, the well-established Korsmeyer–Peppas model was used [56]. Korsmeyer–Peppas categorizes the drug profile based on the release exponent (n) value obtained from the fitting equation. Values of 0.5 or below follow the Fickian diffusion model, whereas values between 0.5 and 0.9 follow a non-Fickian model:

4 $$\frac{{M}_{t}}{{M}_{\infty }}={K}_{kp}{t}^{n}\leftrightarrow \mathrm{log}\left(\frac{{M}_{t}}{{M}_{\infty }}\right)=\mathrm{log}\left({K}_{kp}\right)+n\mathrm{log}(t)$$

where ${M}_{t}$ is the amount of drug released in time t, ${M}_{\infty }$ is the amount of drug released after time $\infty$, n is the drug release exponent and ${K}_{kp}$ is the Korsmeyer release constant of the apparent release.

## Antimicrobial activity

For the zone of inhibition tests, the electrospun mats were punched into 8 mm circular discs weighing approximately 1 mg and disinfected with ultra-violet (UV) radiation for 1 h. The bacterial strains were isolated from single colonies and cultivated in Lysogeny broth (LB) overnight at 37 °C on a rotary shaker. The following day, 100 μL of bacterial solution were spread on Petri dishes containing Mueller-Hintor agar, and two antimicrobial electrospun discs were placed firmly on the surface of each plate and incubated for 24 h at 37 °C. CS and PA6 only fiber discs were also prepared as control groups. Images were taken using a petri dish analyzer (Scan 500, Interscience, France) on the following day, and the radius of inhibition of each plate was measured by ImageJ software.

The optical density (OD) that correlates to the number of living bacteria present within the culture after being exposed to the antimicrobial NFs was evaluated using a spectrophotometer (SynergyMX, BioTek Instruments, USA) at OD_600nm_ absorbance. Pre-cultures of 100 µL *S. aureus* and *P. aeruginosa* deriving from single colonies were prepared in 5 mL Brain heart infusion broth (BHI) and incubated for 37 °C at 160 rpm. The following day, the pre-cultures were suspended to OD_600_ value 0.1 in BHI and incubated for 1 h to obtain exponentially growing cells. After, the culture were resuspended to OD_600_ value 0.05 in 0.2% vol. BHI broth, corresponding to 2.2 × 10^7^ and 1.8 × 10^6^ colony forming units (CFU) per mL of *P. aeruginosa* and *S. aureus*. The electrospun nanofiber mats were placed on the bottom of a 96-well plate in triplicates, disinfected with UV radiation for 1 h; a bacterial solution of 200 µL was added to it, and the sample was incubated for a further 4 h at 37 °C/30 rpm. Growth kinetics were performed by plating on a fresh plate 20 µL of the bacterial supernatant exposed to the antimicrobial scaffolds and 180 µL of fresh BHI. Readings were obtained every 30 min for 24 h at 37 °C at OD_600_. The control was prepared out of 180 µL BHI and 20 µL 0.2% vol% BHI broth.

After incubation of the bacteria having an OD value of 0.1 with the electrospun NFs for 6 h, the bacteria containing scaffolds were assayed with an equal volume mixture of SYTO 9® (3.34 mM, Excitation 483 nm, emission 503 nm) and propidium iodide (PI; 20 mM, Excitation 535 nm, emission 617 nm). The live/dead assessment was then carried out via fluorescence microscopy (Leica DM6000B, Germany), using the 40× objective and the relative fluorescent from the metadata was quantified by measuring the intensity of the mean relative brightness value after subtracting the background signal via ImageJ. The same bacteria-containing scaffolds were fixed in 4% formaldehyde solution for 2 h, gradually dehydrated in ethanol in 1 h intervals (50%, 60%, 70%, 80%, 90%, 95%, 100% v/v) and left to dry in hexamethyldisilazane (HDMS) overnight. The following day, the fixed electrospun mats were sputter-coated with 8 nm gold and imaged by SEM.

All quantitative data are shown as mean ± standard deviation (SD). Statistical significance between the different groups was analyzed by one-way analysis of variance (ANOVA) and Student’s t-test using SPSS v.24 (IBM, USA).

## Supplementary information

**Additioanl file 1: Figure S1.** Schematic representation of the co-axial electrospinning setup. **Figure S2.** Chemical structures of the proposed polymers and antimicrobial system.** Figure S3.** Fiber diameter distribution curves of the electrospun nanofibers. **Figure S4.** STEM micrographs of the core/shell NFs in different flow rates. **Figure S5.** FTIR-ATR spectra of (A) the raw and drug free fibers and (B) antimicrobial substance. **Figure S6.** FTIR-ATR region specific spectra of the drug containing nanofibrous mats. **Figure S7.** XPS survey of the electrospun nanofibrous mats. **Figure S8.** Absorbance spectra of 5CLO8Q and PHMB.** Figure S9.** Standard curves of PHMB and 5CLO8Q at pH 6.2 and 7.2.** Table S1.** XPS analysis of the C1s binding energies.

## Data Availability

All data and material used in this study are available upon request.
