# Supplementary material for: Nylon-6/chitosan core/shell antimicrobial nanofibers for the prevention of mesh-associated surgical site infection
Source: J Nanobiotechnology. 2020 Mar 18;18:51. doi: 10.1186/s12951-020-00602-9 (PMC7081698; doi:10.1186/s12951-020-00602-9)
Supplement: Supplementary file 1 — Additioanl file 1: Figure S1. Schematic representation of the co-axial electrospinning setup. Figure S2. Chemical structures of the proposed polymers and antimicrobial system. Figure S3. Fiber diameter distribution curves of the electrospun nanofibers. Figure S4. STEM micrographs of the core/shell NFs in different flow rates. Figure S5. FTIR-ATR spectra of (A) the raw and drug free fibers and (B) antimicrobial substance. Figure S6. FTIR-ATR region specific spectra of the drug containing nanofibrous mats. Figure S7. XPS survey of the electrospun nanofibrous mats. Figure S8. Absorbance spectra of 5CLO8Q and PHMB. Figure S9. Standard curves of PHMB and 5CLO8Q at pH 6.2 and 7.2. Table S1. XPS analysis of the C1s binding energies. [file 12951_2020_602_MOESM1_ESM.docx]

Supporting Information

**Nylon-6/Chitosan core/shell antimicrobial nanofibers for the prevention of mesh-associated surgical site infection**

Antonios Keirouz^1,2^, Norbert Radacsi^2^, Qun Ren^3^, Alex Dommann^4^, Guido Beldi^5^, René M. Rossi^1^, Giuseppino Fortunato^1*^

**^1^**Empa, Swiss Federal Laboratories for Materials Science and Technology, Laboratory for Biomimetic Membranes and Textiles, St. Gallen, CH-9014, Switzerland.

**^2^**School of Engineering, Institute for Materials and Processes, The University of Edinburgh, Robert Stevenson Road, Edinburgh, EH9 3FB, United Kingdom.

**^3^**Empa, Swiss Federal Laboratories for Materials Science and Technology, Laboratory for Biointerfaces, Bacteria at Surfaces, St. Gallen, CH-9014, Switzerland.

^4^Empa, Swiss Federal Laboratories for Materials Science and Technology, Center for X-ray Analytics, Überlandstrasse 129, Dübendorf, CH-8600, Switzerland.

^5^Department of Visceral Surgery and Medicine, Visceral Surgery, Inselspital University Hospital Bern and University Bern, Bern, Switzerland.

*Corresponding author. Tel.: +41 587657677. E-mail address: giuseppino.fortunato@empa.ch

Table of contents

[Figure S1. Schematic representation of the co-axial electrospinning setup ………………………...…...………...S-3](#_Toc7609338)

[Figure S2. Chemical structures of the proposed polymers and antimicrobial system…………..…...………...S-4](#_Toc7609338)

[Figure S3. Fiber diameter distribution curves of the electrospun nanofibers……………………………………....S-4](#_Toc7609422)

[Figure S4. STEM micrographs of the core/shell NFs in different flow rates……………………………………….…S-5](#_Toc7609422)

[Figure S5. FTIR-ATR spectra of (A) the raw and drug free fibers and (B) antimicrobial substance……….S-6](#_Toc7609423)

[Figure S6. FTIR-ATR region specific spectra of the drug containing nanofibrous mats………………..…..….S-6](#_Toc7609424)

[Table S1. XPS analysis of the C1s binding energies…………………….………………...……………………………………..S-7](#_Toc7609426)

[Figure S7. XPS survey of the electrospun nanofibrous mats……….………………...……………………………………..S-7](#_Toc7609426)

[Figure S8 Absorbance spectra of 5CLO8Q and PHMB…………………………………………………………………………S-8](#_Toc7609420)

[Figure S9. Standard curves of PHMB and 5CLO8Q at pH 6.2 and](#_Toc7609421) 7.2………………………………………..…………S-9


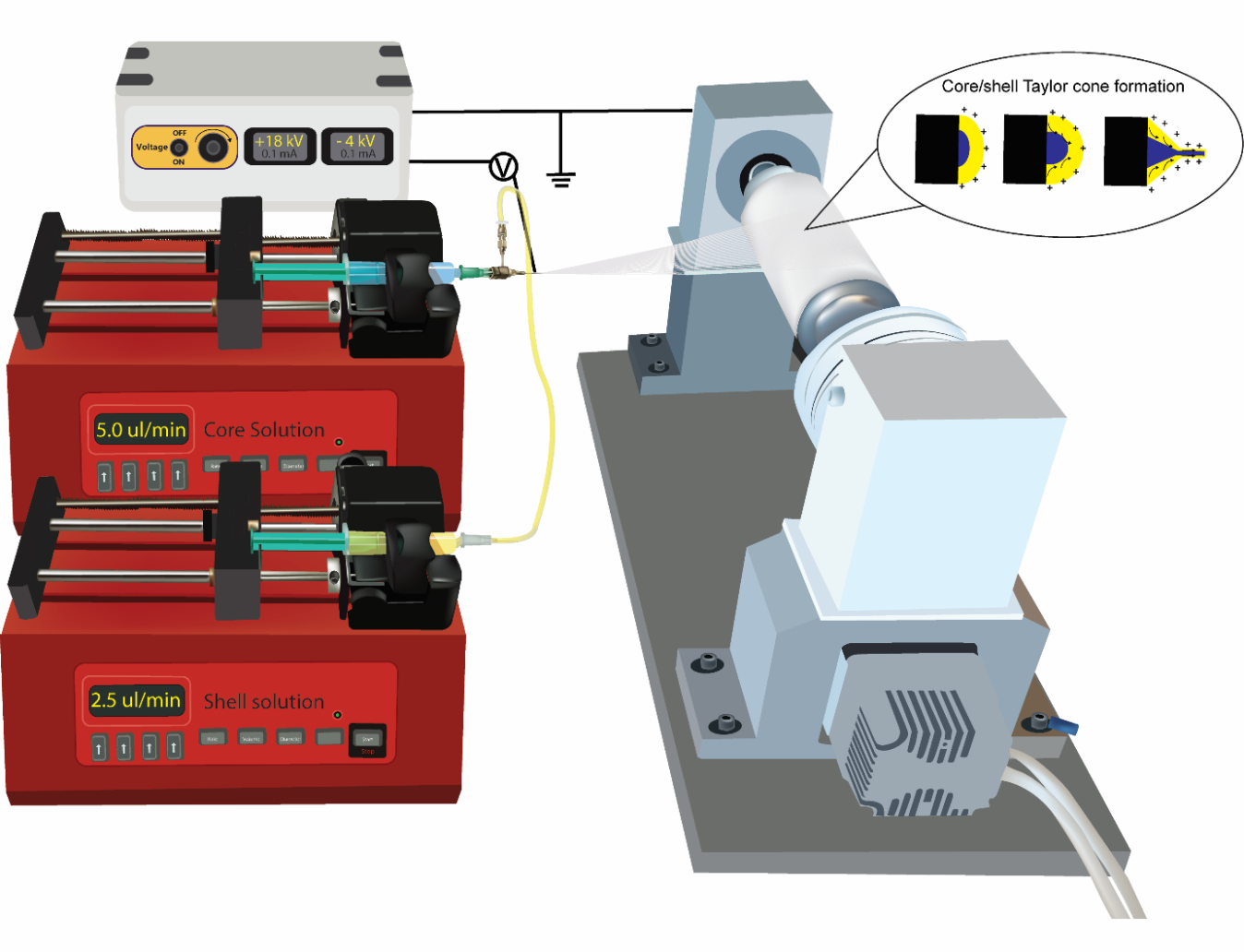


**Figure S1.** Schematic representation of the co-axial electrospinning setup used for the production of core/shell PA6-PHMB/CS-5CLO8Q nanofibrous membranes.

**
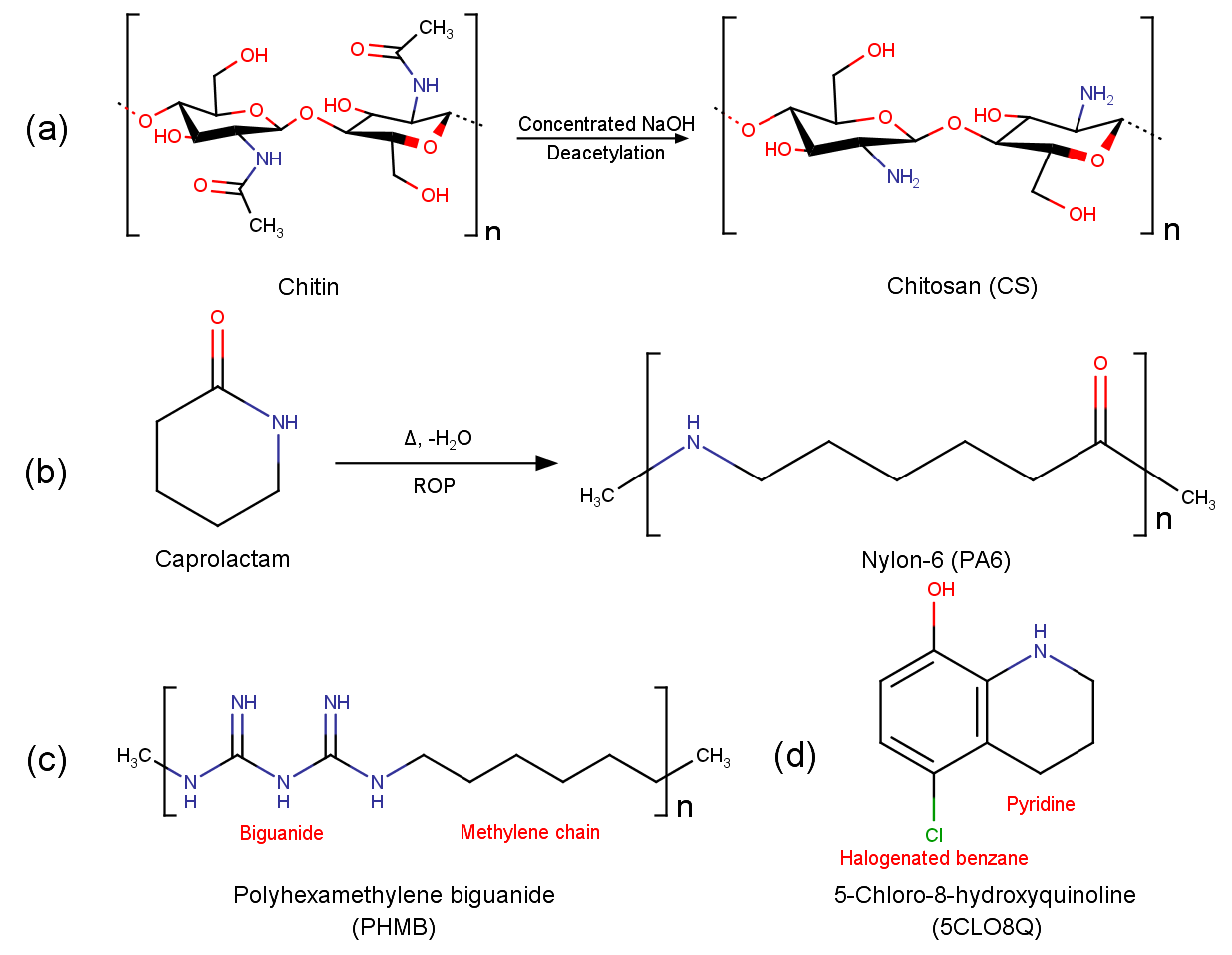
** Figure S2. Chemical structures of the proposed polymers and antimicrobial system. (a) Deacetylation of chitin to yield CS, (b) Ring opening polymerization of ϵ-caprolactam to form PA6, (c) PHMB and (d) 5CLO8Q.

**
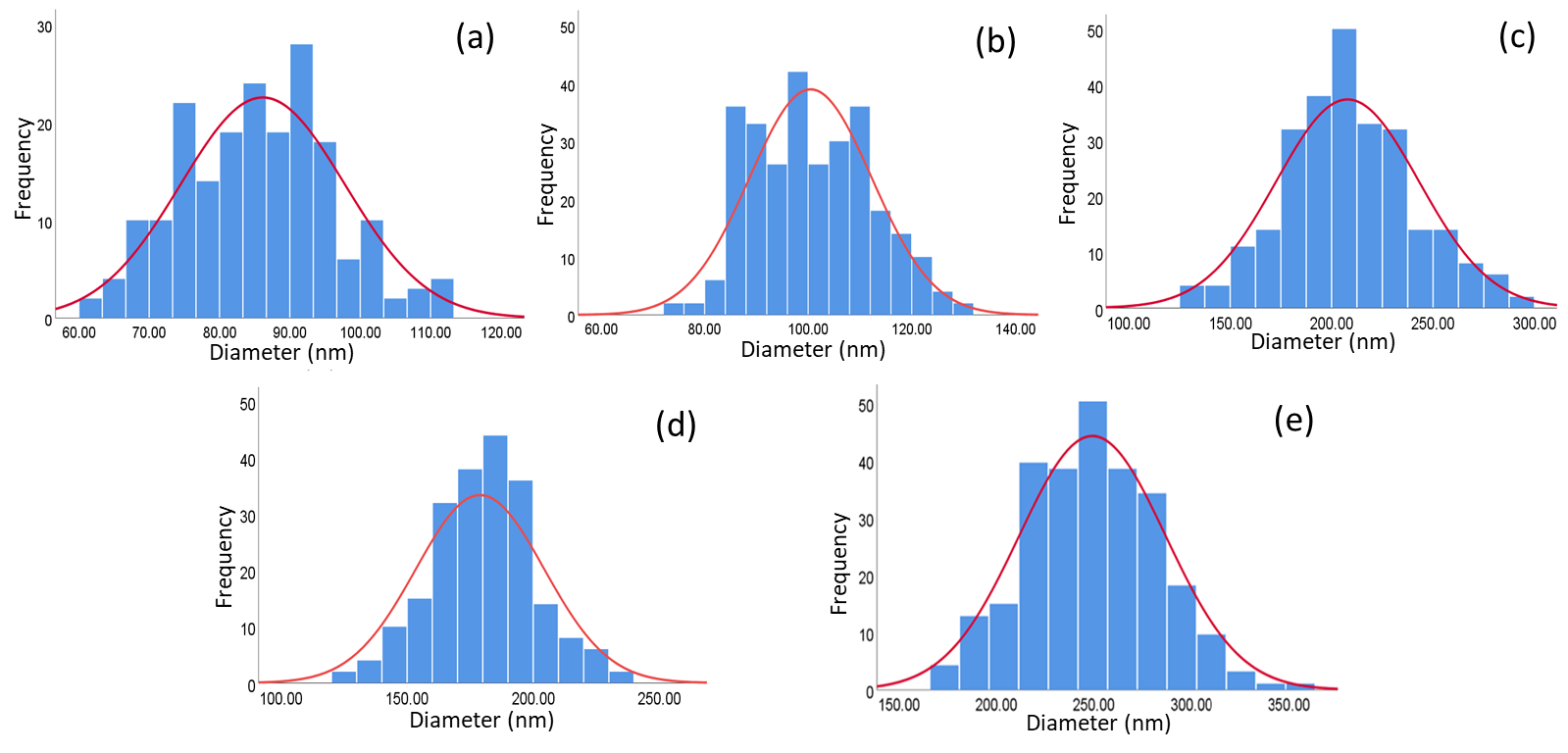
**

Figure S3. Fiber diameter distributions of the electrospun (a) CS, (b) PA6, (c) CS-5CLO8Q, (d) PA6-PHMB and (e) core/shell PA6-PHMB/CS-5CLO8Q nanofibrous mats.

**
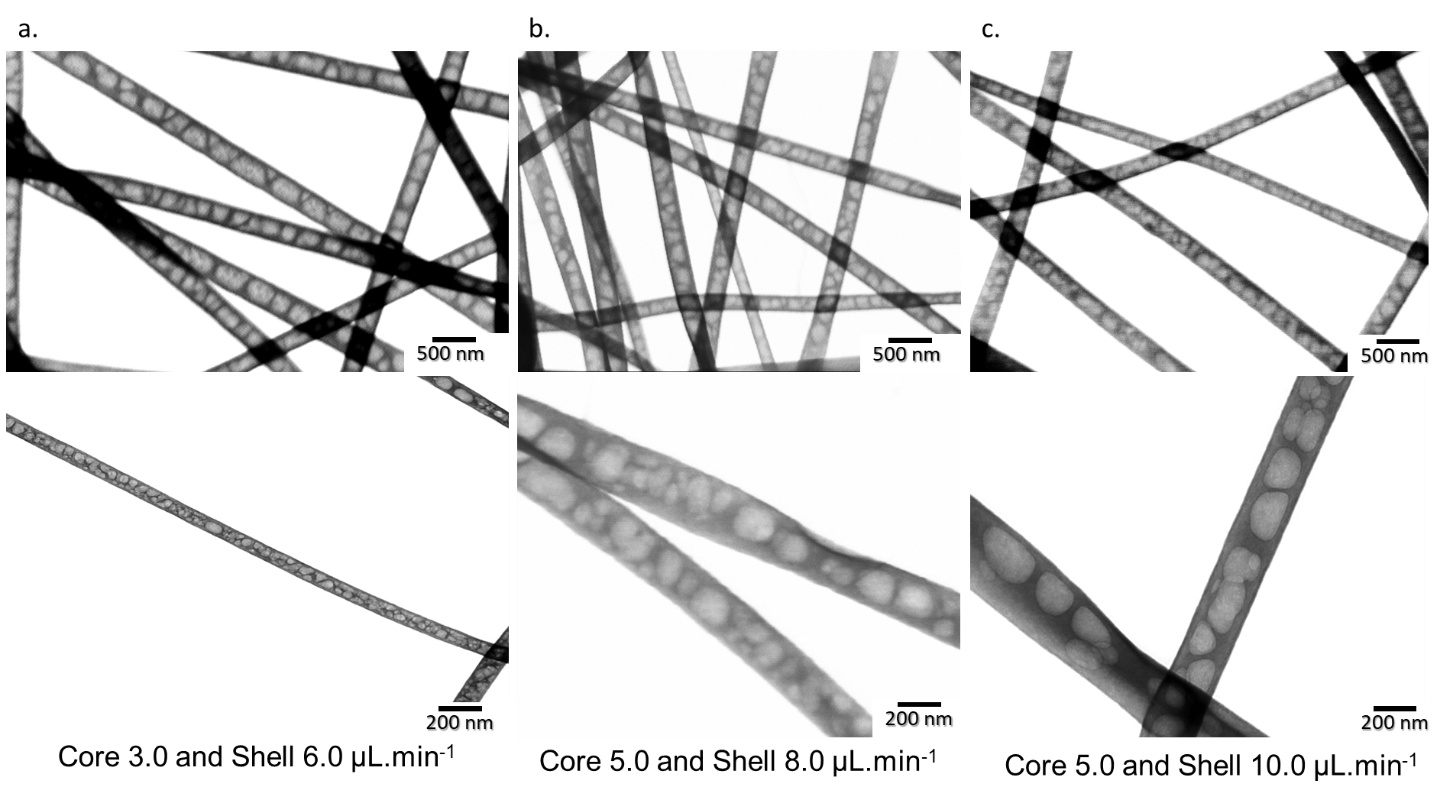
**

Figure S4. STEM micrographs of the PA6-PHMB/CS-5CLO8Q core/shell NFs in different flow rates, under the same electrospinning and solution parameters. (a.) Core to shell feed rate 3.0 and 6.0 , (b.) 5.0 and 8.0 and (c.) 5.0 and 10.0 μL.min^-1^).

**(a)**

**
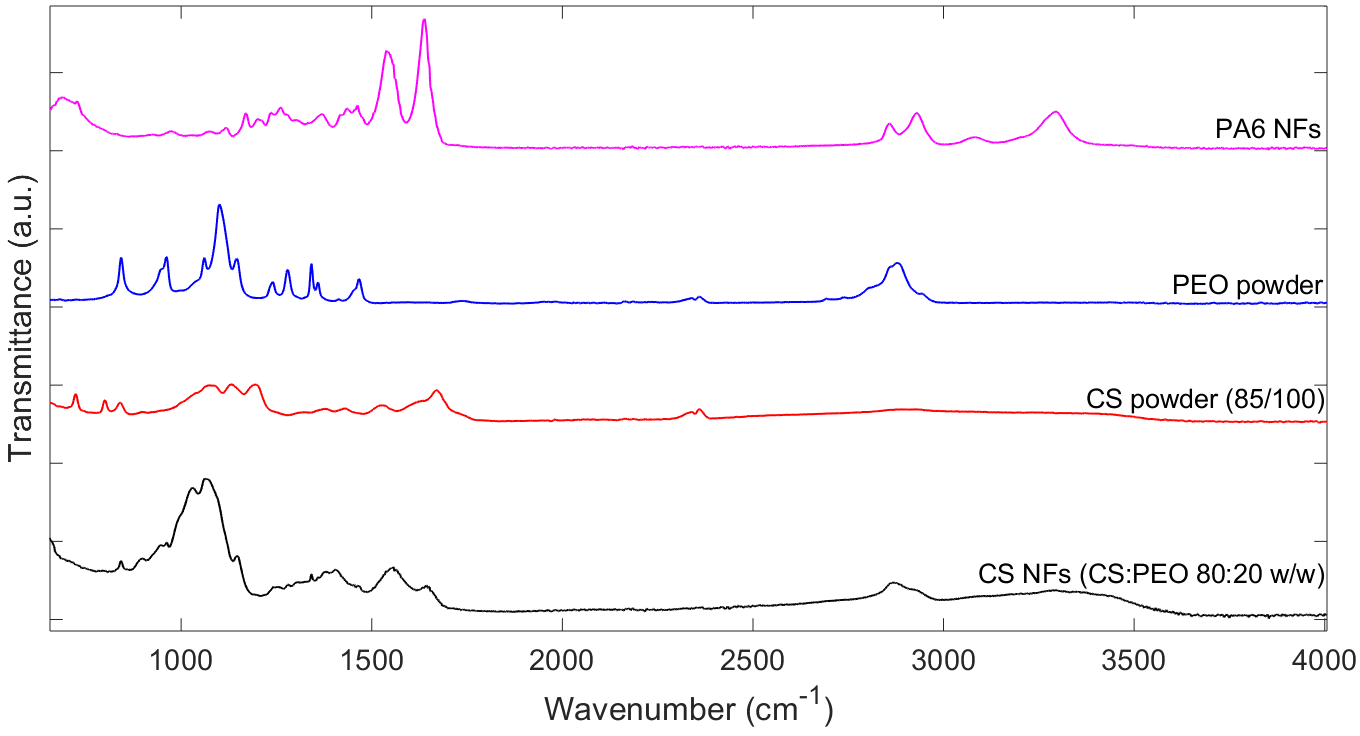
**

**
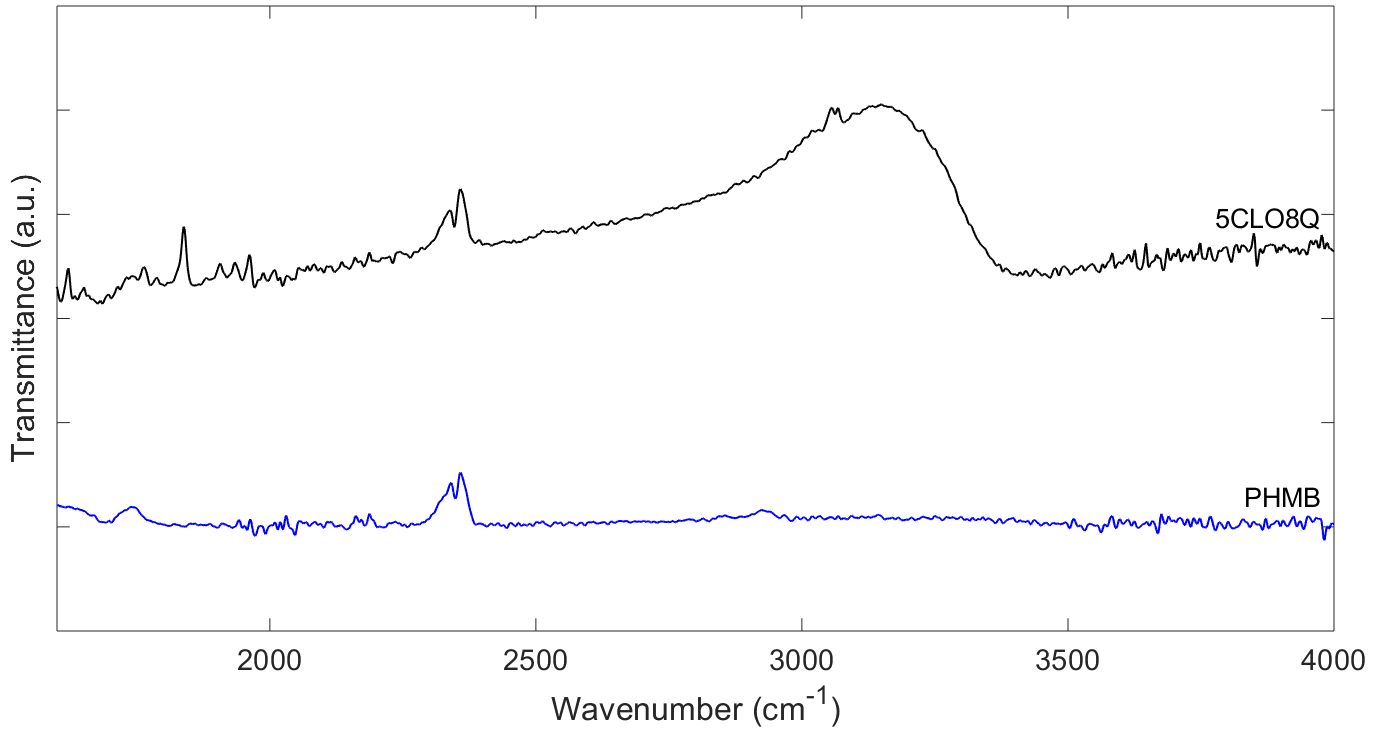
**Figure S5. FTIR-ATR spectra of the produced (a) drug-free 3% w/v CS (CS:PEO 80:20 w/w) (CS) and 21% w/v PA6 NFs, and pristine CS and PEO powders. (b) Pristine antimicrobial substances 5CLO8Q powder and PHMB powder.

**(b)**

**
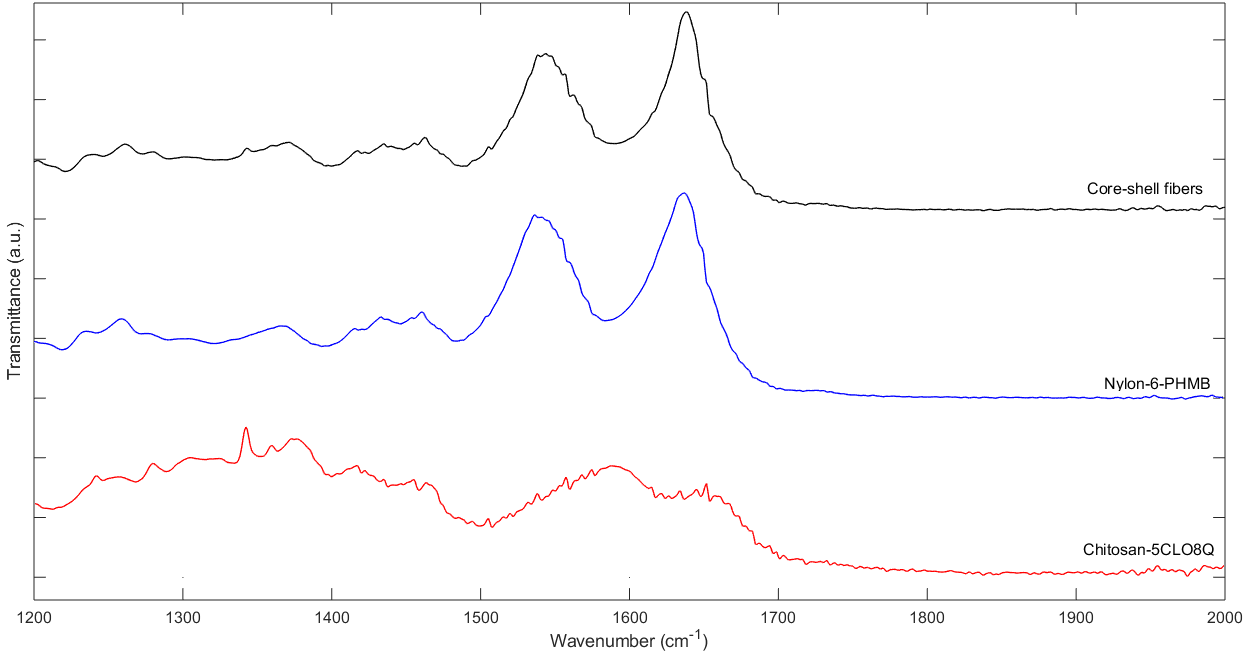
**

Figure S6. FTIR-ATR region specific spectra (1200-2000 cm^-1^) of the drug-containing CS-5CLO8Q, PA6-PHMB, and core/shell NFs.

**Table S1.** XPS analysis of the carbon C1s binding energies.

| **Bending Energy (eV)** | **Group Peaks** | **Chitosan-5CLO8Q** |
| --- | --- | --- |
|  | | **At. Conc. (%)** |
| 284.5 | C-C, C-H | 23.1 |
| 285.5 | C=O | 22.3 |
| 286.5 | C-N, C-O | 54.6 |
| **eV** | **Group** | **Nylon-6-PHMB** |
|  | | **At. Conc. (%)** |
| 284.4 | C-C, C-H | 68.5 |
| 287.3 | C=O | 16.4 |
| 285.4 | C-N | 15.1 |
| **eV** | **Group** | **Core/Shell*** |
|  | | **At. Conc. (%)** |
| 285.7 | C-N, C-O | 47.2 |
| 286.3 | C=O | 25.0 |
| 284.2 | C-C, C-H | 27.8 |

*****Core/shell is PA6-PHMB/CS-5CLO8Q. At. Conc. (%) = atomic concentration (%).

**
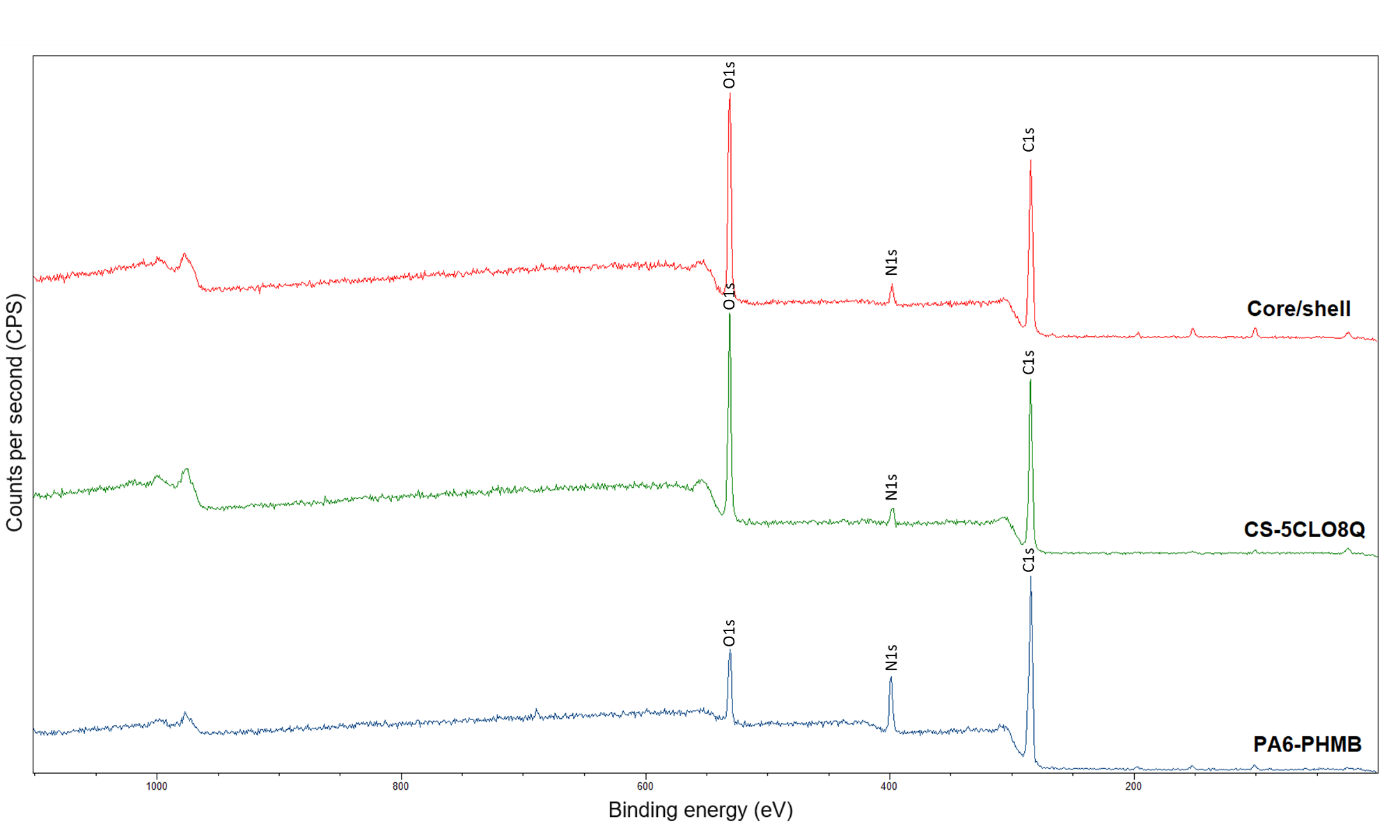
**

Figure S7. XPS survey of the PA6-PHMB, CS-5CLO8Q and core/shell PA6-PGMB/CS-5CLO8Q nanofibrous mats.

**
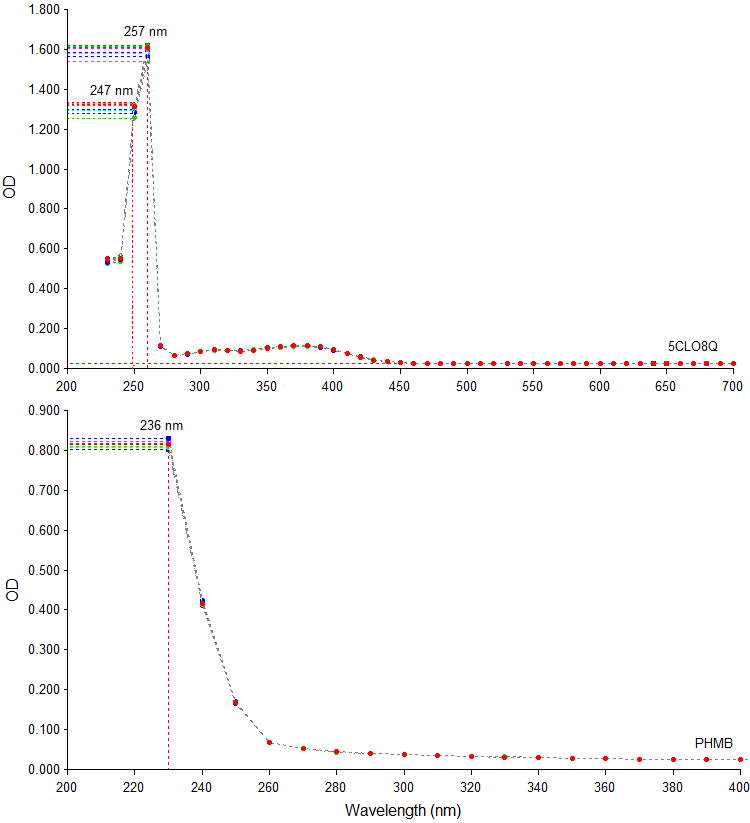
**

Figure S8. Absorbance spectra of 5CLO8Q (top) and PHMB (bottom).

**
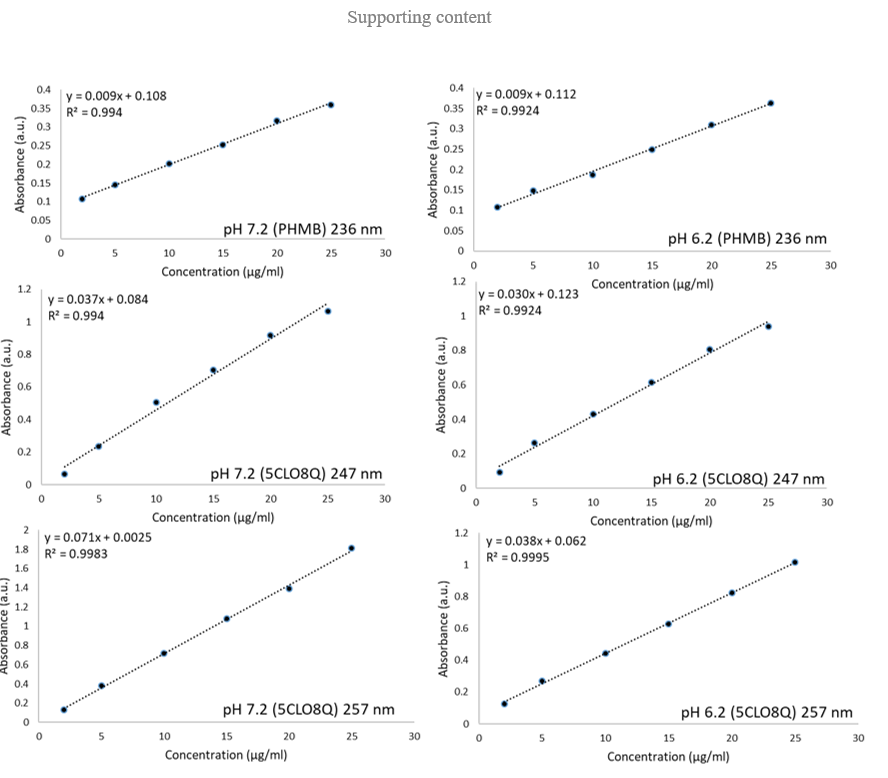
**

Figure S9. Standard curves of PHMB (236 nm) and 5CLO8Q (247 and 257 nm) at pH 7.2 and 6.2.
